# *GRIN2A*-related disorders: genotype and functional consequence predict phenotype

**DOI:** 10.1093/brain/awy304

**Published:** 2018-12-12

**Authors:** Vincent Strehlow, Henrike O Heyne, Danique R M Vlaskamp, Katie F M Marwick, Gabrielle Rudolf, Julitta de Bellescize, Saskia Biskup, Eva H Brilstra, Oebele F Brouwer, Petra M C Callenbach, Julia Hentschel, Edouard Hirsch, Peter C Kind, Cyril Mignot, Konrad Platzer, Patrick Rump, Paul A Skehel, David J A Wyllie, Giles E Hardingham, Conny M A van Ravenswaaij-Arts, Gaetan Lesca, Johannes R Lemke, GRIN2A study group, Alexis Arzimanoglou, Paul B Augustijn, Patrick Van Bogaert, Helene Bourry, Peter Burfeind, Yoyo Chu, Brian Chung, Diane Doummar, Patrick Edery, Aviva Fattal-Valevski, Mélanie Fradin, Marion Gerard, Christa de Geus, Boudewijn Gunning, Danielle Hasaerts, Ingo Helbig, Katherine L Helbig, Rami Jamra, Mélanie Jennesson Lyver, Jolien S Klein Wassink-Ruiter, David A Koolen, Damien Lederer, Roelineke J Lunsing, Mikaël Mathot, Hélène Maurey, Shay Menascu, Anne Michel, Ghayda Mirzaa, Diana Mitter, Hiltrud Muhle, Rikke S Møller, Caroline Nava, Margaret O’Brien, Evelyn van Pinxteren-Nagler, Anne van Riesen, Christelle Rougeot, Damien Sanlaville, Jolanda H Schieving, Steffen Syrbe, Hermine E Veenstra-Knol, Nienke Verbeek, Dorothée Ville, Yvonne J Vos, Pascal Vrielynck, Sabrina Wagner, Sarah Weckhuysen, Marjolein H Willemsen

**Affiliations:** 1 Institute of Human Genetics, University of Leipzig Hospitals and Clinics, Leipzig, Germany; 2 Analytic and Translational Genetics Unit, Massachusetts General Hospital, MA, USA; 3 Program for Medical and Population Genetics/Stanley Center for Psychiatric Research, Broad Institute of Harvard and MIT, Cambridge, MA, USA; 4 University of Groningen, University Medical Centre Groningen, Department of Neurology, Groningen, The Netherlands; 5 University of Groningen, University Medical Centre Groningen, Department of Genetics, Groningen, The Netherlands; 6 Centre for Discovery Brain Sciences, University of Edinburgh, Hugh Robson Building, George Square, Edinburgh, UK; 7 Institut de Génétique et de Biologie Moléculaire et Cellulaire, Illkirch, France, Centre National de la Recherche Scientifique, U7104, Illirch France; 8 Institut National de la Santé et de la Recherche Médicale, U1258, Illkirch, France; 9 Université de Strasbourg, Illkirch, France; 10 Department of Neurology, Strasbourg University Hospital, Strasbourg, France; 11 Department of Pediatric and Clinical Epileptology, Sleep Disorders and Functional Neurology, University Hospitals of Lyon, Lyon, France; 12 CeGaT GmbH and Praxis für Humangenetik, Tübingen, Germany; 13 University Medical Center Utrecht, Department of Genetics, Utrecht, The Netherlands; 14 Medical and Surgical Epilepsy Unit, Hautepierre Hospital, University of Strasbourg, Strasbourg, France; 15 Simons Initiative for the Developing Brain, University of Edinburgh, Hugh Robson Building, George Square, Edinburgh, UK; 16 Centre for Brain Development and Repair, inStem, Bangalore, India; 17 Assistance Publique-Hôpitaux de Paris, Département de Génétique, Groupe Hospitalier Pitié-Salpêtrière, Paris, France; 18 Centre de Référence Déficiences Intellectuelles de Causes Rares, Paris, France; 19 GRC Sorbonne Université “Déficience Intellectuelle et Autisme”, Paris, France; 20 UK Dementia Research Institute at The University of Edinburgh, Edinburgh Medical School, 47 Little France Crescent, Edinburgh, UK; 21 Department of Genetics, Lyon University Hospitals, Lyon, France; 22 Lyon Neuroscience Research Centre, CNRS UMR5292, INSERM U1028, Lyon, France; 23 Claude Bernard Lyon I University, Lyon, France

**Keywords:** channelopathy, molecular genetics, learning disability, childhood epilepsy, spike-wave EEG

## Abstract

Alterations of the *N*-methyl-d-aspartate receptor (NMDAR) subunit GluN2A, encoded by *GRIN2A*, have been associated with a spectrum of neurodevelopmental disorders with prominent speech-related features, and epilepsy. We performed a comprehensive assessment of phenotypes with a standardized questionnaire in 92 previously unreported individuals with *GRIN2A*-related disorders. Applying the criteria of the American College of Medical Genetics and Genomics to all published variants yielded 156 additional cases with pathogenic or likely pathogenic variants in *GRIN2A*, resulting in a total of 248 individuals. The phenotypic spectrum ranged from normal or near-normal development with mild epilepsy and speech delay/apraxia to severe developmental and epileptic encephalopathy, often within the epilepsy-aphasia spectrum. We found that pathogenic missense variants in transmembrane and linker domains (mis_TMD+Linker_) were associated with severe developmental phenotypes, whereas missense variants within amino terminal or ligand-binding domains (mis_ATD+LBD_) and null variants led to less severe developmental phenotypes, which we confirmed in a discovery (*P* = 10^−6^) as well as validation cohort (*P* = 0.0003). Other phenotypes such as MRI abnormalities and epilepsy types were also significantly different between the two groups. Notably, this was paralleled by electrophysiology data, where mis_TMD+Linker_ predominantly led to NMDAR gain-of-function, while mis_ATD+LBD_ exclusively caused NMDAR loss-of-function. With respect to null variants, we show that *Grin2a*^+/−^ cortical rat neurons also had reduced NMDAR function and there was no evidence of previously postulated compensatory overexpression of GluN2B. We demonstrate that null variants and mis_ATD+LBD_ of *GRIN2A* do not only share the same clinical spectrum (i.e. milder phenotypes), but also result in similar electrophysiological consequences (loss-of-function) opposing those of mis_TMD+Linker_ (severe phenotypes; predominantly gain-of-function). This new pathomechanistic model may ultimately help in predicting phenotype severity as well as eligibility for potential precision medicine approaches in *GRIN2A*-related disorders.

## Teaser

Strehlow *et al.* describe the largest cohort to date of individuals with GRIN2A-related disorders. The results reveal two phenotypic subgroups associated with different classes of variants affecting distinct domains of the GluN2A protein with different functional consequences. The findings will help predict outcomes in newly diagnosed individuals.

## Introduction

*N*-methyl-d-aspartate receptors (NMDAR) are expressed throughout the brain, mediating excitatory neurotransmission important for development, learning, memory, and other higher cognitive functions. NMDAR are di- or tri-heterotetrameric ligand-gated ion channels composed of two glycine-binding GluN1 (encoded by *GRIN1*) and two glutamate-binding GluN2 subunits (*GRIN2A–D*) (Traynelis *et al.*, 2010). All GluN subunits are composed of an extracellular, a transmembrane and an intracellular component. The extracellular component consists of the amino-terminal domain (ATD) with binding sites for antagonists such as Zn^2+^ and the ligand-binding domains (LBD) S1 and S2 specific for agonist binding including glycine and glutamate. The channel pore is formed by the three transmembrane domains (TMD), M1, M3, M4, and a re-entrant pore-loop M2. The C-terminal domain (CTD) is involved in mediating signals within the intracellular compartment. Compared with the ubiquitously expressed GluN1 subunit, the GluN2 subunits show specific spatiotemporal expression profiles throughout the CNS (Paoletti *et al.*, 2013). Whereas GluN2B and GluN2D subunits are predominantly expressed prenatally, expression of GluN2A and GluN2C is low prenatally but significantly increases shortly after birth (Bar-Shira *et al.*, 2015).

Four genes encoding NMDAR subunits (*GRIN1*, *GRIN2A*, *GRIN2B*, and *GRIN2D*) have so far been linked to human disease; *GRIN2A* appears to be associated with the broadest and best characterized phenotypic spectrum, including a variety of disorders of the epilepsy aphasia spectrum and developmental and epileptic encephalopathy, such as Landau-Kleffner syndrome and epileptic encephalopathy with continuous spike-and-wave during slow-wave sleep (CSWS) ([Bibr awy304-B13][Bibr awy304-B14]; Carvill *et al.*, 2013).

*GRIN2A *is a gene with a significantly reduced number of missense variants in controls compared to the expected number of variants in a similarly sized gene (missense z-score 3.8) (Lek *et al.*, 2016). The ratio of 31.2 expected versus 3 observed null variants in ExAC and the probability of loss-of-function intolerance of 1.00 (pLI score) suggests that *GRIN2A *null and missense variants strongly reduce evolutionary fitness (Lek *et al.*, 2016).

Investigation of functional consequences of disease-associated *GRIN2A *missense variants revealed various gain- or loss-of-function effects (Endele *et al.*, 2010; Lesca *et al.*, 2013; Pierson *et al.*, 2014; Swanger *et al.*, 2016; Addis *et al.*, 2017; Chen *et al.*, 2017; Sibarov *et al.*, 2017). Identification of null variants likely leading to GluN2A haploinsufficiency further complicated understanding of underlying pathomechanisms. GluN2A haploinsufficiency is expected to cause reduced expression of GluN2A, which was thought to potentially be compensated for by consecutive upregulation of expression of other GluN subunits, especially GluN2B, leading to an altered NMDAR assembly (Balu and Coyle, 2011). NMDAR containing GluN2B have slower deactivation times than those containing GluN2A (Paoletti *et al.*, 2013; Wyllie *et al.*, 2013). Replacement of GluN2A by GluN2B has thus been hypothesized to increase the duration of activation of the NMDAR suggesting a net gain-of-function effect mediated by *GRIN2A* null variants.

To delineate the phenotypic spectrum of *GRIN2A*-related disorders, we reviewed previously reported and newly identified individuals with pathogenic or likely pathogenic variants in *GRIN2A.* We provide a comprehensive phenotypic dataset of 248 individuals with variants in *GRIN2A* and integrate these data with protein domain and electrophysiological data. Specifically, we aimed at elucidating genetic and functional correlates to the wide phenotypic range of *GRIN2A*-related disorders, for which we reviewed published electrophysiological data and investigated consequences of *Grin2a* knock-out in cortical rat neurons.

## Materials and methods

### Cohort recruitment

Data on 92 previously unreported individuals with (likely) pathogenic *GRIN2A* variants (ENST00000396573) were collected from several diagnostic and research cohorts. Clinical and genetic information were obtained with a specific questionnaire tailored to phenotypes previously reported in individuals with *GRIN2A *variants (Supplementary Table 1). We also ascertained additional, more detailed phenotypic information on individuals that had previously been published. All information about the listed variants has been added to an open-access online database (www.grin-database.de).

### Review of the literature and variant classification

We searched the literature (www.ncbi.nlm.nih.gov/pubmed) (up to 23 March 2018) for reports of cases with *GRIN2A* variants and reviewed the associated clinical and genetic information. This study has been approved by the ethics committee of the University of Leipzig (224/16-ek, 402/16-ek).

Based on the recommendations of the American College of Medical Genetics and Genomics (ACMG) (Richards *et al.*, 2015; Nykamp *et al.*, 2017), we classified missense variants fulfilling at least one of the following conditions (in addition to constraint and prediction scores) as likely pathogenic:

*de novo* + absent from controls*

*OR* confirmative functional studies + absent from controls*

*OR de novo* + confirmative functional studies

*OR* present in three or more affected and no healthy individuals of one family + absent from controls*

*OR* novel missense variant at a location that had been classified as pathogenic according to the above conditions + absent from controls*.

Furthermore, null variants located in exon 3–14 (until amino acid position 838) were classified as (likely) pathogenic.

*Controls were over 120 000 people without severe paediatric disease compiled in the gnomAD browser [genome Aggregation Database (http://gnomad.broadinstitute.org/)]. Only variants classified as pathogenic or likely pathogenic were considered for further genotype-phenotype correlations in this study, regardless of the associated phenotype.

### Statistical analysis

All statistical analyses were done with the R programming language (www.r-project.org). Fisher’s exact test for Count Data, Wilcoxon rank-sum test and Cochran Armitage test were performed as referenced in the ‘Results’ section. *P*-values were corrected for multiple testing with the Bonferroni method. For Fisher’s exact test, we reported odds ratios (OR) and 95% confidence intervals (95% CI). To investigate variant clustering in different phenotypes, we calculated the distance (linear amino acid sequence) of all possible variant pairs of individuals with the same intellectual disability/developmental delay (ID/DD) phenotypes to all combinations of different ID/DD phenotypes (mild versus severe). We compared the variant distances of same versus different phenotypes with Wilcoxon rank-sum tests. The R code used to perform the statistical analyses and figures is available upon request.

### Ranking severity of intellectual disability/developmental delay

We identified 178 individuals with detailed information about the presence or absence of ID/DD and apportioned categories reflecting the severity of the phenotype according to the Diagnostic and Statistical Manual of Mental Disorders (DSM-5): no ID/DD (0 points), mild ID/DD (1 point), moderate ID/DD (2 points), severe ID/DD (3 points), and profound ID/DD (4 points) (Supplementary Table 4). The terms ID and DD are used interchangeably here.

### Neuronal culture, generation of the *Grin2a*^−/−^ rat and RNA quantification

Cortical rat neurons were cultured as described (Baxter *et al.*, 2011) at a density of between 9–13 × 10^4^ neurons per cm^2 ^from embryonic Day 20.5 rats with Neurobasal^TM^ growth medium supplemented with B27 (Invitrogen). Experiments were performed at days *in vitro* (DIV) 7–16, as indicated.

To generate the *Grin2a*^−/−^ rat, single cell Long Evans Hooded rat embryos underwent pronuclear microinjection of mRNA encoding the enzyme Cas9 and small guide RNAs (sgRNA) binding to the 5′ and 3′ end of exon 8 of *Grin2a*, before being implanted into pseudopregnant mothers. The resulting live births were screened by polymerase chain reaction (PCR) for genomic deletions due to repair by non-homologous end joining of double-stranded breaks targeted to either side of exon 8. A 1065 bp deletion spanning exon 8 (which encodes key pore forming domains of GluN2A) was identified, and confirmed by sequencing (data not shown). Genotyping was carried out using primer pairs P1 (AGGGAAGAAGGGAACAGGAG) with P2 (TCTCTGGGATTCAGTGCAGA) and P3 (AAGGCAGAGAGAGAGACAAAG) with P4 (ATGGCAGTTCCCAGTAGCAT). P1 and P3 bind to the 5′ end of the deletion, P2 binds to the 3′ end of the deletion, and P4 binds within the deletion. The sgRNA design and generation of the founder animals was performed by Horizon Discovery Group plc. All the experiments were performed using wild-type, heterozygous, and homozygous littermate matched animals. Animals were treated and all experiments performed in accordance with UK Animal Scientific Procedures Act (1986) following local ethical review.

RNA was isolated from cultured neurons using the Roche High Pure RNA Isolation Kit (including DNase treatment), according to the manufacturer’s instructions (Roche). Three wells from a 24-well plate were pooled for each animal. cDNA was synthesized from 13 µg RNA using a Transcriptor First Strand cDNA Synthesis Kit (Roche), according to the manufacturer’s instructions, then stored at −20°C. For real time PCR (RT-PCR), cDNA was diluted to the equivalent of 6 ng of initial RNA per 15 µl qPCR reaction, per gene of interest. RT-PCR was performed in a Stratagene Mx3000P QPCR System (Agilent Technologies), using the FS universal SYBR Green MasterRox mix (Roche), according to the manufacturer’s instructions. The required amount of template was mixed with water, SYBR Green MasterRox mix and forward and reverse primers (200 nM each final concentration) to the required reaction volume. Primers used were: *Grin2a*: AGCCAGAGACCCCGCTAC and TGGGGTGCACCTGGTAAC; *Gadph*: AGAAGGCTGGGGCTCACC and AGTTGGTGGTGCAGGATGC. Technical replicates as well as no template and no reverse transcription negative controls were included. The quantitative reverse transcriptase (qRT)-PCR cycling programme was 10 min at 95°C, then 40 cycles of 30 s at 95°C, 40 s at 60°C, with detection of fluorescence and 1 min at 72°C, followed by one cycle (for dissociation curve) of 1 min at 95°C, and 30 s at 55°C, with a ramp up to 30 s at 95°C, (ramp rate: 0.2°C/s) with continuous detection of fluorescence on the 55–95°C ramp. Data were normalized to *Gadph* expression.

### Cell culture electrophysiological recording and analysis

Coverslips containing cortical neurons were transferred to a recording chamber perfused (at a flow rate of 3–5 ml/min) with an external recording solution composed of (in mM): 150 NaCl, 2.8 KCl, 10 HEPES, 2 CaCl_2_, 10 glucose and 0.1 glycine, pH 7.3 (320–330 mOsm). Tetrodotoxin (300 nM) was included to block action-potential driven excitatory events. Patch pipettes were made from thick-walled borosilicate glass (Harvard Apparatus) and filled with a K-gluconate-based internal solution containing (in mM): potassium gluconate 141, NaCl 2.5, HEPES 10, EGTA 11; pH 7.3 with KOH. Electrode tips were fire-polished for a final resistance ranging between 3–5 MΩ. All NMDAR currents were evoked by 150 µM NMDA and 100 µM glycine, both applied using a perfusion system. Currents were recorded at room temperature (21 ± 2°C) using an Axopatch 200B amplifier (Molecular Devices). Neurons were voltage-clamped at −65 mV and recordings were rejected if the holding current was >−100 pA or if the series resistance drifted by >20% of its initial value (<20 MΩ). Whole-cell currents were analysed using WinEDR v3.2 software (John Dempster, University of Strathclyde, UK). To determine the ifenprodil sensitivity of neurons, whole-cell NMDAR currents were recorded followed by the inclusion of 3 µM ifenprodil in the recording solution for a blocking period of 90 s. The whole-cell NMDAR current was reassessed with 3 µM ifenprodil included, and the % block was calculated. To determine spermine potentiation, neurons were voltage-clamped at −30 mV and switched to a low sodium recording solution composed of (in mM): 70 NaCl, 60 choline chloride, 2.8 KCl, 20 HEPES, 10 glucose, 0.1 glycine, 0.1 diethylenetriaminepentaacetic acid, pH 6.5 with NaOH. NMDA currents were evoked by 150 µM NMDA then reassessed in the presence of 100 µM spermine. Only cells with NMDA-evoked currents >40 pA were included.

### Western blotting

Neurons were lysed in 1.5× lithium dodecyl sulphate sample buffer (NuPage, Life Technologies) and boiled at 100°C for 10 min. Approximately 10 µg of protein was loaded onto a precast gradient gel (4–12%) and subjected to electrophoresis. Briefly, western blotting onto a polyvinylidene fluoride (PVDF) membrane was then carried out using the Xcell SureLock™ system (Invitrogen) according to the manufacturer’s instructions. Following the protein transfer, the PVDF membranes were blocked for 1 h at room temperature with 5% (w/v) non-fat dried milk in Tris-buffered saline with 0.1% Tween 20. The membranes were incubated at 4°C overnight with the primary antibodies diluted in blocking solution: anti-GluN2A (N-terminus, 1:1000, Invitrogen), and anti-beta actin (1:200 000, Abcam) or anti-GluN2B C-terminus (1:8000, BD Biosciences) and anti-beta actin. For visualization of western blots, horseradish peroxidase-based secondary antibodies were used followed by chemiluminescent detection on Kodak® X-Omat film.

### Data availability

The authors confirm that the data supporting the findings of this study are available within the article and/or its Supplementary material.

## Results

We reviewed data on 92 unpublished individuals with (likely) pathogenic *GRIN2A* variants with systemically assessed phenotypes. After re-evaluation of all published *GRIN2A* variants based on ACMG recommendations (Richards *et al.*, 2015; Nykamp *et al.*, 2017), we additionally included 156 previously reported individuals with (likely) pathogenic variants. Thus, we were able to collectively review genotypes and phenotypes of 248 individuals with *GRIN2A*-related disorders.

### The cohort

In our cohort, 45.1% of individuals whose gender was known were female (*n* = 87) and 54.9% were male (*n* = 106). Gender was unknown in 55 cases. The youngest individual was 11 months at evaluation, the oldest 71 years (median 8 years). Of the 248 individuals, 121 (121/248; 48.8%) were single cases, including 65 individuals (65/121; 53.7%) where a *de novo* confirmation of the variant was performed. The remaining 127 individuals (127/248; 51.2%) were found in 36 different families.

Among 3038 individuals with neurodevelopmental disorders with epilepsy screened by epilepsy panel sequencing (covering *GRIN2A*) in the same diagnostic lab, seven displayed (likely) pathogenic variants revealing a prevalence of 0.23% in this disease spectrum.

### Variant type and distribution

**Figure 1:**
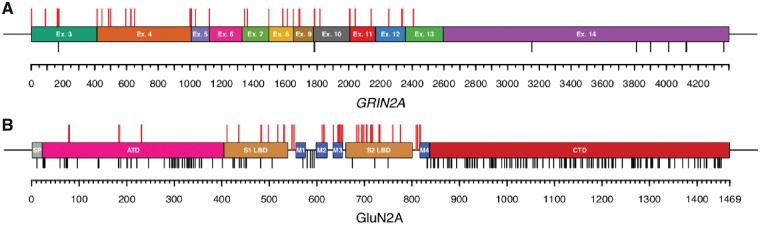
**Distribution of variants. **(**A**) Pathogenic or likely pathogenic null variants (red bars) are spread over nearly the entire gene. However, according to ACMG criteria, the last exon 14 is spared, which encodes nearly the complete C-terminal domain. Null variants in healthy gnomAD controls (black bars) occur primarily in the last exon 14 (probability loss-of-function intolerance 1.00 in ExAC). (**B**) Pathogenic or likely pathogenic missense variants (red bars) cluster in regions of *GRIN2A* encoding functionally important domains (S1 and S2 ligand binding domains as well as M1–M4 transmembrane domains and linker regions). The density of missense variants in healthy gnomAD controls (MAC = 2, black bars) is highest in the intracellular C-terminal domain.

One hundred and forty-five individuals (145/248; 58.5%) had likely protein-truncating variants referred to as null variants including 35 individuals (35/145; 24.1%) with nonsense variants, 23 individuals (23/145; 15.9%) with small frameshift deletions or duplications, 42 individuals (42/145; 29.0%) with canonical splice-site variants, three individuals (3/145; 2.1%) with a loss-of-start codon, 37 individuals (37/145; 25.5%) with gross deletions or duplications spanning up to the whole gene but not affecting adjacent genes and five individuals (5/145; 3.4%) with complex chromosomal rearrangements disrupting *GRIN2A* (Fig. 1A). A total of 53 different null variants were considered (likely) pathogenic, of which 22 were recurrent.

The remaining 103 individuals (103/248; 41.5%) had missense variants, including 13 individuals (13/103; 12.6%) with variants in the extracellular amino-terminal domain and 56 individuals (56/103; 54.4%) with variants in the extracellular ligand-binding domain S1 or S2, all of which are referred to as mis_ATD+LBD_ (for protein domains, see Supplementary Table 5). In addition, six individuals (6/103; 5.8%) had variants in linker regions and 28 individuals (28/103; 27.2%) had variants in the three transmembrane domains, M2–M4, referred to as mis_TMD+Linker_. No variants affecting the C-terminus met ACMG criteria of being (likely) pathogenic (Fig. 1B). A total of 44 different missense variants were considered (likely) pathogenic, of which 23 were recurrent.

### Intellectual disability/developmental delay

**Figure 2:**
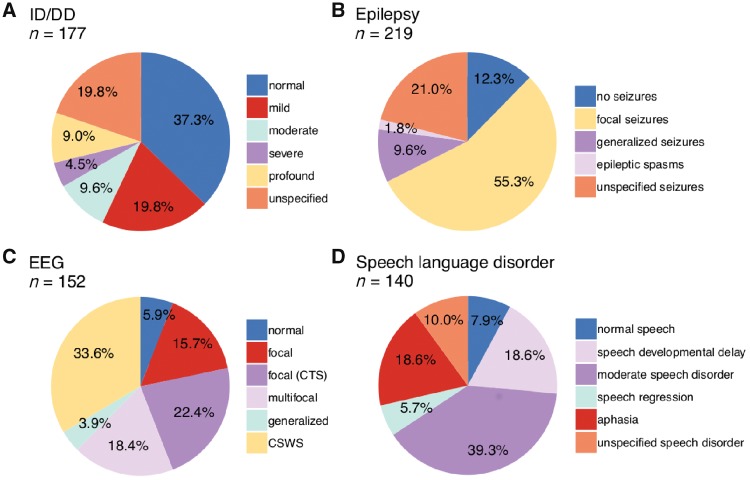
**Distribution of phenotypes. **Individuals with *GRIN2A*-related disorders display a broad range of phenotype severity and expressivity with respect to (**A**) intellectual outcome, (**B**) epilepsy, (**C**) EEG patterns, and (**D**) speech or language impairments.

In our *GRIN2A* cohort, cognitive assessment was available on 177 individuals, of which 111 (111/177; 62.7%) had ID/DD. In 35 cases (35/177; 19.8%), the severity of ID/DD could not be specified in more detail. Among the 177 individuals, the level of ID/DD was mild in 35 cases (35/177; 19.8%), moderate in 17 (17/177; 9.6%), severe in eight (8/177; 4.5%) and profound in 16 (16/177; 9.0%). Sixty-six individuals (66/177; 37.3%) had normal intelligence (Fig. 2A).

### Seizures and electroencephalography

Information on the epilepsy phenotype was available in 219 cases. The majority of patients (192/219; 87.7%) had seizures, including 121 individuals (121/219; 55.3%) with focal seizures (with or without evolution to bilateral tonic-clonic seizures). Twenty-one individuals (21/219; 9.6%) had tonic-clonic seizures of unknown onset, four (4/219; 1.8%) epileptic spasms and 46 cases (46/219; 21.0%) had unspecified seizures. Several individuals displayed a spectrum of different seizure types. Twenty-seven individuals (27/219; 12.3%) did not have seizures (Fig. 2B).

EEG information was available in 152 individuals, including 143 individuals (143/152; 94.1%) displaying epileptiform discharges. In 86 cases (86/152; 56.6%), focal discharges were recorded, of whom 34 cases (34/152; 22.4%) had centrotemporal spikes and 28 cases (28/152; 18.4%) had multifocal discharges. Fifty-one individuals (51/152; 33.6%) had CSWS and six individuals (6/152; 3.9%) had generalized discharges. Only nine individuals (9/152; 5.9%) had a normal EEG (Fig. 2C).

Recognizable epilepsy syndromes comprised the known spectrum of *GRIN2A*-associated epilepsy syndromes, such as benign epilepsy with centrotemporal spikes, atypical childhood epilepsy with centrotemporal spikes and Landau-Kleffner syndrome.

### Language and speech disorders

Information about speech phenotypes was available in 140 cases. The vast majority of individuals presented with speech disorders (129/140; 92.1%). In 115 patients where the type of speech disorder was defined, 55 individuals (55/140; 39.3%) had moderate speech/language impairment including dysarthria, speech dyspraxia, dysphasia, speech regression with residual impairments, sometimes supplemented by minor impairments such as impaired pitch, hypernasality or imprecise articulation. Twenty-six individuals (26/140; 18.6%) had aphasia (including speech regression with loss of speech) and 26 (26/140; 18.6%) had isolated delay of speech development. Eight individuals (8/140; 5.7%) presented with temporary speech regression. The type of speech disorder was not further specified in 14 individuals (14/140; 10.0%). Only 11 individuals (11/140; 7.9%) had normal speech development. Speech disorders were not necessarily linked to EEG abnormalities as 10/11 individuals with normal speech development had abnormal EEG and eight of nine individuals with normal EEG still had abnormal speech development (only one individual had normal speech, normal EEG, no epilepsy, no ID/DD) (Fig. 2D).

### Other neurological and psychiatric phenotypes

Information about tone was available in 139 cases. Forty (40/139; 28.8%) individuals had hypotonia, including 18 individuals (18/139; 13.0%) with no further specification. Among the remaining 22 individuals, 16 (16/139; 11.5%) had mild, two had moderate (2/139; 1.4%) and four individuals (4/139; 2.9%) had severe hypotonia (including one individual with arthrogryposis). Ninety-nine individuals (99/139; 71.2%) had no hypotonia (Supplementary Fig. 2A). We identified 19 (19/72; 26.4%) individuals with movement disorders including ataxia (*n* = 10), dystonic/spastic/choreatic movement disorders (*n* = 8), including two individuals with complex movement disorders (*n* = 2; Individual 039: no ambulation, spasticity, sometimes dystonic, choreatic, athethotic movements; Individual 058: involuntary movements, paroxysmal dyskinesia, movement abnormality of the tongue, abnormality of eye movement, impaired smooth pursuit), and an unspecified movement disorder (*n* = 1) (Supplementary Fig. 2B). Information about neuropsychiatric comorbidities was available in 70 cases. Seventeen individuals (17/70; 24.3%) displayed behavioural or psychiatric disorders, such as attention deficit hyperactivity disorder (*n* = 6), autism spectrum disorder (*n* = 6), schizophrenia (*n* = 2) and anxiety disorder (*n* = 1). Two individuals had unspecified behavioural abnormalities.

### MRI

Brain MRI data were available for 85 individuals. Approximately 14% (12/85; 14.1%) had brain abnormalities, comprising a variety of findings including focal cortical dysplasia, dysplastic corpus callosum with delayed myelinisation, hypoplasia of corpus callosum with midline lipoma, hippocampal hyperintensity, hippocampal sclerosis, heterotopia, subcortical lesion, hypoplastic olfactory bulb, cerebellar glioma, enlarged Virchow-Robin spaces, delayed myelinization (*n* = 1 for each). An additional 11% of individuals (9/85; 10.6%) had generalized volume loss compatible with brain atrophy. Abnormal gyral pattern similar to some cases with *GRIN1*- and *GRIN2B*-related disorders was not observed (Platzer *et al.*, 2017; Fry *et al.*, 2018) and was also not expected in *GRIN2A*-related disorders as knockdown of only GluN1 and GluN2B (but not GluN2A) have been shown to slow down neuronal migration (Jiang *et al.*, 2015). Sixty-four individuals (64/85; 75.3%) had no MRI abnormalities (Supplementary Fig. 2C).

### Genotype–phenotype correlations reveal two distinct phenotype groups

For 177 of all 248 individuals with (likely) pathogenic variants in *GRIN2A*, we obtained detailed information about presence or absence of ID/DD and ranked severity of intellectual disability into five categories (refer to the ‘Materials and methods’ section).

**Figure 3:**
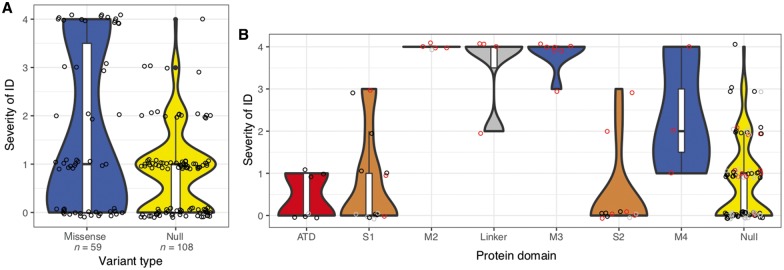
**Severity of ID/DD.** Comparison of severity of ID/DD in carriers of variants in different protein domains. (**A**) Missense (blue) and null (truncating) variants (yellow). (**B**) Missense variants in different protein domains in the order of the linear amino acid sequence and truncating variants (*far right*). Here, variants that were inherited are coloured black, *de novo* variants are red and unknown variants are grey. Violins are plotted to have the same maximum width. *Bottom*, *middle* and *top* of boxplots within violins show the 1st, 2nd and 3rd quartiles of the data; whiskers maximally extend to 1.5× interquartile range.

Comparing 70 individuals with missense and 107 with null variants, we found more severe ID/DD in carriers of missense variants (Cochran Armitage Test, *P*-value = 0.00011). However, individuals with missense variants displayed a bimodal distribution of ID/DD severity (Fig. 3A). We compared spatial variant clustering in individuals with same severity of ID/DD compared to variant clustering in individuals with mixed severity of ID/DD (Wilcoxon Rank test, comparing severe to mixed ID/DD cases *P*-value = 2 × 10^−6^, comparing mild to mixed ID/DD cases: *P*-value = 0.5) suggesting missense variants in different parts of the protein lead to distinct ID/DD phenotypes. We observed that 19 individuals with mis_TMD+Linker_ had more severe phenotypes than 33 individuals with mis_ATD+LBD_ (Fig. 3B). To test this observation statistically, we randomly separated missense carriers into a discovery cohort (*n* = 35) and a validation cohort (*n* = 17). In the discovery cohort (Cochran Armitage Test, *P* = 10^−6^) as well as the validation cohort (Cochran Armitage Test, *P* = 0.0003), carriers of mis_TMD+Linker_ had significantly more severe ranked ID/DD (median_discovery_ 4, median_validation _4) than carriers of mis_ATD+LBD _(median_discovery_ 0, median_validation _0). Accordingly, 32 of 32 mis_TMD+Linker_ were *de novo*, while 18 of 47 mis_ATD+LBD_ were *de novo* (Fisher’s exact test, OR Inf, 95% CI 11 to Inf, *P*-value = 2 × 10^−9^). Other variants were inherited; unknown variants were excluded from the test.

Notably, carriers of the 107 null variants had a similar degree of ID (median 1, mild ID) compared to carriers of mis_ATD+LBD_ (median 0 corresponding to no ID, Cochran Armitage Test, *P*-value = 0.3). Furthermore, all 66 individuals with normal intellect were carriers of mis_ATD+LBD_ or null variants.

**Figure 4:**
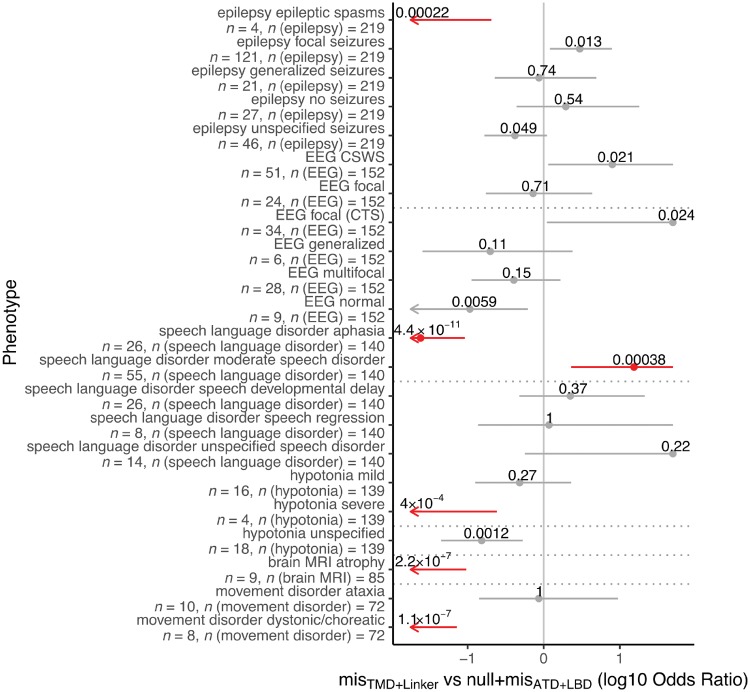
**Phenotypes correlated with protein domains. **Comparison of phenotypes associated with variants in different protein domains (Fisher’s exact test). Phenotype differences being significant after Bonferroni multiple testing correction for 17 × 2 tests are labelled red. For each phenotype, OR with 95% CI (grey/red bars) and number of patients with the phenotype and number of patients for whom the phenotype was assessed are shown. For clarity, OR and CIs are cut at ±1.7. (**A**) Comparison mis_ATD+LBD_ or null variants with mis_TMD+Linker_. CSWS = continuous spike-and-wave during slow-wave sleep; CTS = centrotemporal spikes.

We found significant differences for other phenotypes only between individuals with mis_TMD+Linker_ and those with mis_ATD+LBD_ or null variants but not between those with mis_ATD+LBD_ and null variants (Fig. 4, all phenotype comparisons in Fig. 4 were done with Fisher’s exact test). Although we observed no difference for presence of epilepsy in individuals with mis_TMD+Linker_ and individuals with mis_ATD+LBD_ or null variants (Fisher’s exact test, *P*-value = 0.54), we found significant differences with respect to seizure type as epileptic spasms were only observed in individuals with mis_TMD+Linker_, but not in individuals with mis_ATD+LBD_ or null variants (Fisher’s exact test, *P*-value = 2.6 × 10^−6^). There were also significantly more cases with focal seizures in the cohort with mis_ATD+LBD_/null variants than in the mis_TMD+Linker_ cohort (Fisher’s exact test, *P*-value = 4.1 × 10^−4^, OR 5.0, 95% CI 1.9 to 15.7). There were no significant differences for generalized seizures (Fisher’s exact test, *P*-value = 1.0) or for particular EEG patterns. All individuals with generalized volume loss on MRI were carriers of mis_TMD+Linker_ (Fisher’s exact test, *P*-value = 0.002, OR 5.8, 95% CI 1.7 to 21.3), while this feature was not observed in any carrier of mis_ATD+LBD_/null variants.

### Variance of ID/DD phenotype in individuals with the same genetic variant

**Figure 5:**
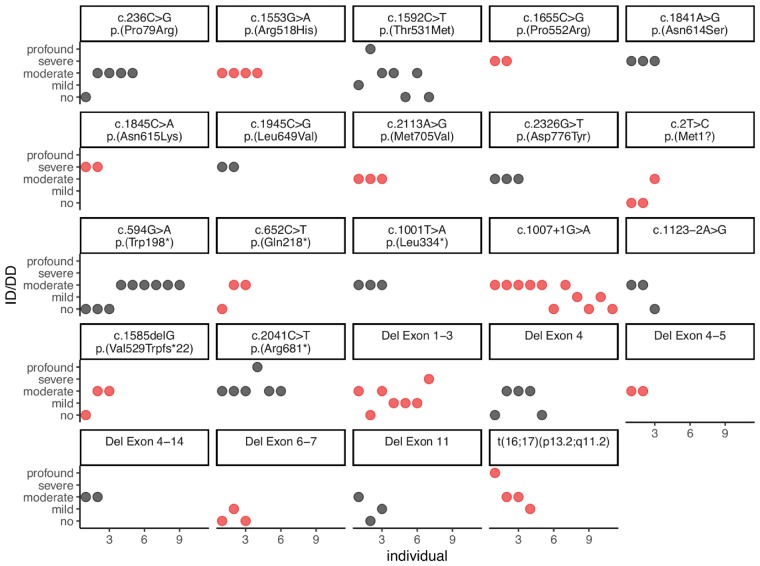
**Variance of ID/DD phenotype in individuals with the same genetic variant.** ID/DD phenotypes (*y*-axis) of all recurrent *GRIN2A* genetic variants are of similar degree suggesting that the same variant leads to similar ID/DD phenotypes despite considerable phenotype expressivity.

We investigated whether individuals with the same genetic variant had similar ID/DD phenotypes (see ‘Materials and methods’ section for classification). We investigated 98 individuals carrying 24 unique variants where ID/DD phenotypes were available in at least two individuals per variant (Fig. 5). The mean variance of ID/DD phenotypes per variant was 0.65 (±0.64 standard deviation, SD). Permuting family labels 10 000 times, we found that the real value was lower than the mean variance in 15 of 10 000 permutations (Supplementary Fig. 2, empirical *P*-value = 0.0016). This suggests that while considerable phenotype expressivity exists, the same variant leads to similar ID/DD phenotypes. However, more and better ID/DD data (e.g. measured as IQ) is needed to optimally study phenotype expressivity.

### Both phenotype groups correspond to opposing electrophysiological consequences

Of the 44 (likely) pathogenic missense variants included in this study (ATD: *n* = 4, LBD: *n* = 20, TMD: *n* = 16, Linker: *n* = 4), 23 (equalling 52%) had been functionally investigated previously (ATD: *n* = 3, LBD: *n* = 14, TMD2–4: *n* = 1 each, Linker: *n* = 3). We compared the published functional data of all 23 missense variants (Supplementary Table 3) (Endele *et al.*, 2010; Lesca *et al.*, 2013; Pierson *et al.*, 2014; Serraz *et al.*, 2016; Swanger *et al.*, 2016; Addis *et al.*, 2017; Chen *et al.*, 2017; Ogden *et al.*, 2017; Sibarov *et al.*, 2017; XiangWei *et al.*, 2018). The 23 variants comprised six mis_TMD+Linker_ variants displaying predominantly gain-of-function effects (5× gain-of-function versus 1× loss-of-function), while all 17 extracellular mis_ATD+LBD_ variants show exclusively loss-of-function activity (Fisher’s exact test, *P*-value = 2 × 10^−4^, OR Inf, 95% CI 5.3 to Inf). We conclude that these opposing electrophysiological consequences are the most likely explanation for the significantly different degree of severity of ID/DD as well as other phenotypic differences associated with mis_ATD+LBD_ and mis_TMD+Linker_. The currently single exception to this pattern, the loss-of-function mis_TMD+Linker_ variant c.1642G>A, p.(Ala548Thr), was found in an individual with moderate ID. In total, 32 of 34 individuals with mis_TMD+Linker_ had *de novo* variants, while two were of unknown origin. On the other hand, the majority of the variants in other regions were inherited.

### Rat model and electrophysiological analysis

**Figure 6:**
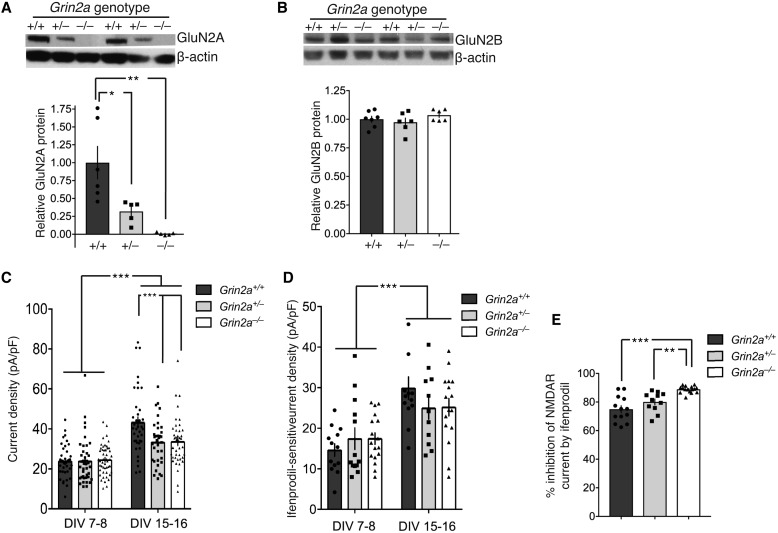
**No compensation by GluN2B. **GluN2B-mediated currents do not increase to compensate for GluN2A deficiency in cortical neurons from a *Grin2a* knock-out rat. (**A**) Western blot and quantification confirming the absence of GluN2A expression in *Grin2a*^−/−^ neurons, and an intermediate expression level in *Grin2a*^+/−^ neurons at 15 DIV. Tukey’s test reveals a significant difference between *Grin2a*^+/+^ versus *Grin2a*^+/−^ (*P* = 0.0196) and versus *Grin2a*^−/−^ (*P* = 0.0014). *Grin2a*^+/+^: *n* = 6; *Grin2a*^+/−^: *n* = 5; *Grin2a*^−/−^: *n* = 5. (**B**) Western blot and quantification confirming no changes in GluN2B expression in either *Grin2a*^+/−^ or *Grin2*^−/−^ neurons compared to *Grin2a*^+/+^ neurons at 15 DIV. *Grin2a*^+/+^, *n* = 6; *Grin2a*^+/−^: *n* = 5; *Grin2a*^−/−^: *n* = 5. (**C**) NMDA (150 µM) evoked currents were measured in cortical neurons of the indicated genotypes and periods of culture. Currents were calculated and normalized to cell capacitance to give a value for the current density within the neuron. Two-way ANOVA reports a significant developmental stage effect (*P* < 0.0001) and a significant genotype effect (*P* = 0.013) as well as a significant interaction between the two (*P* = 0.0059). Sidak’s *post hoc* test reveals a significant difference between *Grin2a*^+/+^ versus *Grin2a*^+/−^ (*P* = 0.0007) and versus *Grin2a*^−/−^ (*P* = 0.0006). *Grin2a*^+/+^: *n* = 38 (7–8 DIV), 38 (15–16 DIV) cells, eight animals; *Grin2a*^+/−^: *n* = 40 (7–8 DIV), 35 (15–16 DIV) cells, nine animals; *Grin2a*^−/−^: *n* = 48 (7–8 DIV), 31 (15–16 DIV) cells, 10 animals. (**D**) NMDA (150 µM) evoked currents were measured in cortical neurons of the indicated genotypes and periods of culture before and after the application of the GluN2B-selective antagonist ifenprodil (3 µM). The ifenprodil-sensitive current was calculated and normalized to cell capacitance. Two-way ANOVA reports a significant developmental stage effect (*P* < 0.0001) but no significant genotype effect (*P* = 0.880) nor a significant interaction between the two (*P* = 0.154). *Grin2a*^+/+^: *n* = 13 (7–8 DIV), 13 (15–16 DIV) cells, four animals; *Grin2a*^+/−^: *n* = 13 (7–8 DIV), 11 (15–16 DIV) cells, five animals; *Grin2a*^−/−^: *n* = 17 (7–8 DIV), 16 (15–16 DIV) cells, five animals. (**E**) At 15–16 DIV the percentage inhibition of NMDA (150 µM) evoked currents by ifenprodil (3 µM) was significantly greater (Tukey’s test) in *Grin2a*^−/−^ neurons compared to *Grin2a*^+/+^ neurons (*P < *0.0001) and *Grin2a*^+/−^ neurons (*P* = 0.0038). DIV = day *in vitro*.

Our phenotypic data suggest that the clinical consequences of *GRIN2A* null variants are similar to the clinical consequences of mis_ATD+LBD_ loss-of-function variants. However, it has previously been hypothesized that *GRIN2A* null variants could ultimately result in NMDAR with gain-of-function (or altered function) through compensatory increased expression of other NMDAR subunits, particularly *GRIN2B.* We therefore sought to determine whether homozygous or heterozygous loss of *Grin2a* results in compensatory upregulation of *Grin2b* expression. We used a newly created *Grin2a* knockout rat, and compared NMDAR currents in cortical neurons cultured from these rats with those from their wild-type and heterozygous littermates (litters generated by Het-Het crosses) (Fig. 6A). In this model, there is no detectable compensatory increase in the expression level of GluN2B protein (Fig. 6B). We analysed NMDAR currents at two developmental stages: after 7–8 DIV when currents are almost exclusively GluN2B-dominated, and at 15–16 DIV when there is a significant proportion of GluN2A-containing NMDARs (Supplementary Fig. 3) (Edman *et al.*, 2012). Analysis of NMDAR current density at 7–8 DIV revealed no genotype-dependent difference, consistent with the near-exclusive presence of GluN2B-containing diheteromeric NMDARs (Fig. 6C). Analysis of currents at 15–16 DIV showed an age-dependent increase of currents, as expected, but also a deficit in currents in *Grin2a*^+/−^ and *Grin2a*^−/−^ neurons, relative to wild-type. This suggests that any compensation of GluN2A deficiency through an increase in other NMDAR subunits is insufficient to rescue currents to wild-type levels.

We next investigated whether there was any evidence of compensation through GluN2B upregulation that could be detected via electrophysiological assessment. If there was compensation, then the magnitude of whole cell currents dependent on GluN2B would be expected to be higher in *Grin2a*^+/−^ and *Grin2a*^−/−^ neurons, relative to wild-type, at 15–16 DIV. We measured the portion of the whole cell currents sensitive to the GluN2B-selective antagonist ifenprodil (Fig. 6D), but found no difference in the magnitude of ifenprodil-sensitive current at 15–16 DIV (or 7–8 DIV). Nevertheless, the percentage of total NMDAR currents sensitive to ifenprodil block was increased in *Grin2a*^−/−^ neurons as would be expected for neurons where GluN2A expression is absent (Fig. 6E). Thus, within this experimental system, there appears to be no evidence for increases in GluN2B expression to compensate for loss of GluN2A expression due to *Grin2a* allelic deletion.

## Discussion

We present a comprehensive investigation of *GRIN2A*-related phenotypes, comprising 248 affected individuals with pathogenic or likely pathogenic variants in *GRIN2A.*

### Variant distribution

We observed a clustering of disease-causing missense variants in the highly conserved ligand-binding domains S1 and S2 as well as transmembrane and linker domains, which is similar to our previous observations in *GRIN1* and *GRIN2B* (Lemke *et al.*, 2016; Platzer *et al.*, 2017) and may assist in predicting pathogenicity of variants of uncertain significance by its location (Fig. 1). No missense variants in the intracellular C-terminal domain of GluN2A (beyond amino acid position 838) have been found to fulfil ACMG criteria for being pathogenic or likely pathogenic. Previous reports of alleged disease-associated C-terminal variants may therefore be revised, as this region is also the only region in *GRIN2A* that shows no evidence of regional depletion as the number of observed variants in ExAC was not higher than expected by a mutational model (Lek *et al.*, 2016; Samocha, 2017), similar to GluN1 and GluN2B (Lemke *et al.*, 2016; Platzer *et al.*, 2017). As the C-terminus of GluN2A is tolerant to genetic variation in the general population, we conclude that most missense variants in the C-terminus likely have no effects.

### Phenotypic range

Our comprehensive analysis shows that the *GRIN2A*-related phenotypic spectrum does not only comprise well established epilepsy-aphasia disorders, but is much broader and ranges from normal or near-normal development to non-specific developmental and epileptic encephalopathy. Notably, only three individuals had an apparently normal phenotype with no ID, no epilepsy and no speech disorder (two of them also had EEG investigation, both with normal result), and all were relatives of more severely affected individuals. Moreover, five individuals with (likely) pathogenic variants are listed in gnomAD and can therefore also be considered normal, even though very minor phenotypic abnormalities cannot be excluded. Thus, reduced penetrance appears to be possible but not a common phenomenon among carriers of pathogenic or likely pathogenic *GRIN2A* variants. Epilepsy and speech disorders seen in >80% of individuals occur independent of intellectual disability, which is present in 62.7% of individuals and was mild in nearly half of those cases. This is in stark contrast to phenotypes related to *GRIN1*, *GRIN2B* and *GRIN2D* that are associated with marked ID in nearly 100% of cases (Lemke *et al.*, 2016; Li *et al.*, 2016; Platzer *et al.*, 2017). Among all currently known GRIN-associated phenotypes, *GRIN2A*-related disorders display the most recognizable epilepsy spectrum, comprising focal or multifocal epilepsy with or without centrotemporal spikes as well as CSWS (Fig. 2). As normal and near-normal development are part of the phenotypic range, it can be assumed that individuals with milder phenotypes are more likely to pass on their pathogenic variants, which may explain why 60.2% of variants of known origin are inherited and do not exclusively occur *de novo*, as is the rule for disorders related to *GRIN1*, *GRIN2B* and *GRIN2D* (Lemke *et al.*, 2016; Li *et al.*, 2016; Platzer *et al.*, 2017).

### Genotype–phenotype correlation

In contrast to previous studies (Myers and Scheffer, 1993), our systematic analyses of phenotype and molecular data of a large cohort of individuals with *GRIN2A* variants identified two distinct phenotype groups corresponding to the location of variants in different protein domains (Fig. 4). Mis_TMD+Linker_ are associated with severe developmental and epileptic encephalopathy phenotypes, whereas mis_ATD+LBD_ are associated with speech abnormalities and/or seizures with mild to no ID only. Strikingly, both phenotypic groups are significantly correlated with opposing electrophysiological consequences of the NMDAR, even though the complex functional alterations caused by a *GRIN2A* variant cannot always easily be reduced to a binary description such as loss- or gain-of-function. It appears plausible that mis_LBD_ may impede agonist binding and thus reduce channel activity, whereas a mis_TMD+Linker _may affect formation of the ion channel pore mediating a gain-of-function effect by e.g. disrupted channel inhibition by Mg^2+^ (Pierson *et al.*, 2014; Swanger *et al.*, 2016; Addis *et al.*, 2017; Chen *et al.*, 2017). However, more electrophysiology data of mutated NMDAR are needed to clarify exact pathomechanisms of variants in the different protein domains.

### Pathomechanistic model

We observed that individuals with extracellular mis_ATD+LBD_ (displaying exclusively loss-of-function effects) have a comparable phenotypic range to individuals with null variants, which is substantially less severe than the phenotypes of individuals with membrane-associated mis_TMD+Linker_ (displaying predominantly gain-of-function effects). We therefore hypothesize that loss-of-function mis_ATD+LBD_ and null variants mediate similar pathomechanistic effects.

In agreement with our phenotype data but in contrast to previous hypotheses, we observed that *Grin2a^−^^/+^* and *Grin2a^−^^/^^−^* cultured rat neurons show lower current density, indicating that any compensatory increase in expression of other GluN subunits is not sufficient to match the current normally mediated by GluN2A-containing NMDAR in rats. Furthermore, application of the GluN2B-specific blocker ifenprodil to these neurons did not give any evidence of compensatory increase of GluN2B in NMDAR assembly in GluN2A-deficient cells. Our data thus contradict the hypothesis of a compensatory gain-of-function effect due to GluN2A haploinsufficiency and in fact suggest loss-of-function, in agreement with our phenotype-based observations. Namely, *GRIN2A* null variants are associated with comparable clinical consequences as mis_ATD+LBD_ (resulting in loss-of-function) and with markedly less severe clinical consequences than mis_TMD+Linker _(resulting predominantly in gain-of-function). With our pathomechanistic model, we predict that individuals with developmental and epileptic encephalopathy due to mis_TMD+Linker_ are prone to having an underlying gain of NMDAR function and represent promising candidates for treatment with NMDAR blockers, such as memantine (Pierson *et al.*, 2014). However, currently there are still little data available on clinical treatment of *GRIN2A*-related disorders with memantine (Pierson *et al.*, 2014). Conversely, individuals with variants leading to complete or partial loss of channel function (mis_ATD+LBD_ or null variants) may potentially respond to positive allosteric modulators of the NMDAR (Zhu and Paoletti, 2015; Addis *et al.*, 2017).

Our study illustrates how systematically investigating clinical phenotypes in a large cohort of individuals with a monogenic disease cannot only reveal novel genotype-phenotype correlations, but also contribute to a better understanding of the underlying functional mechanisms being a prerequisite for the development of precision medicine approaches.

## Supplementary Material

Supplementary Data

## Abbreviations

ACMG: American College of Medical Genetics and Genomics
ATD: amino-terminal domain
DD: developmental delay
ID: intellectual disability
LBD: ligand-binding domain
NMDAR: *N*-methyl-d-aspartate receptors
TMD: transmembrane domain

## Appendix 1

### *GRIN2A* study group

Full affiliation details are available in the Supplementary material.

Alexis Arzimanoglou, Paul B. Augustijn, Patrick Van Bogaert, Helene Bourry, Peter Burfeind, Yoyo Chu, Brian Chung, Diane Doummar, Patrick Edery, Aviva Fattal-Valevski, Mélanie Fradin, Marion Gerard, Christa de Geus, Boudewijn Gunning, Danielle Hasaerts, Ingo Helbig, Katherine L. Helbig, Rami Jamra, Mélanie Jennesson Lyver, Jolien S. Klein Wassink-Ruiter, David A. Koolen, Damien Lederer, Roelineke J. Lunsing, Mikaël Mathot, Hélène Maurey, Shay Menascu, Anne Michel, Ghayda Mirzaa, Diana Mitter, Hiltrud Muhle, Rikke S. Møller, Caroline Nava, Margaret O’Brien, Evelyn van Pinxteren-Nagler, Anne van Riesen, Christelle Rougeot, Damien Sanlaville, Jolanda H. Schieving, Steffen Syrbe, Hermine E. Veenstra-Knol, Nienke Verbeek, Dorothée Ville, Yvonne J. Vos, Pascal Vrielynck, Sabrina Wagner, Sarah Weckhuysen, Marjolein H. Willemsen.
