# Supplementary material for: GRIN2A-related disorders: genotype and functional consequence predict phenotype
Source: Brain. 2018 Dec 12;142(1):80–92. doi: 10.1093/brain/awy304 (PMC6308310; doi:10.1093/brain/awy304)
Supplement: Supplementary Data [file awy304_supp.zip › awy304-suppl_data/brain-2018-01122-File013.pdf]

DNA	Protein	domain	Glutamate	Glycine	Membrane expression	Magnesium	Proton	Zinc	Deactivation Rate	Amplitude, Peak	Calcium	result	reference
c.236C>G	p.(Pro79Arg)	ATD	decreased potency	decreased potency	reduced	NA	NA	NA	NA	NA	NA	LoF	Addis L, Virdee JK, Vidler LR, Collier DA, Pal DK, Ursu D.: Epilepsy-associated GRIN2A mutations reduce NMDA receptor trafficking and agonist potency – molecular profiling and functional rescue. Sci Rep 2017, 7(1):66.
c.551T>G	p.(Ile184Ser)	ATD	NA	NA	reduced	NA	NA	NA	increased	decreased	NA	LoF	Sibarov DA, Bruneau N, Antonov SM, Szepetowski P, Burnashev N and Giniatullin R (2017) Functional Properties of Human NMDA Receptors Associated with Epilepsy-Related Mutations of GluN2A Subunit. Front. Cell. Neurosci. 11:155.
c.551T>G	p.(Ile184Ser)	ATD	no effect	no effect	no effect	NA	no effect	no effect	no effect	NA	NA	NO	Serraz B, Grand T, Paoletti P.: Altered zinc sensitivity of NMDA receptors harboring clinically-relevant mutations. Neuropharmacology 2016, 109:196-204.
c.692G>A	p.(Cys231Tyr)	ATD	decreased potency	decreased potency	reduced	NA	NA	NA	NA	decreased	NA	LoF	Addis L, Virdee JK, Vidler LR, Collier DA, Pal DK, Ursu D.: Epilepsy-associated GRIN2A mutations reduce NMDA receptor trafficking and agonist potency – molecular profiling and functional rescue. Sci Rep 2017, 7(1):66.
c.1306T>C	p.(Cys436Arg)	S1	no response	no response	no expression	NA	NA	NA	NA	NA	NA	LoF	Addis L, Virdee JK, Vidler LR, Collier DA, Pal DK, Ursu D.: Epilepsy-associated GRIN2A mutations reduce NMDA receptor trafficking and agonist potency – molecular profiling and functional rescue. Sci Rep 2017, 7(1):66.
c.1306T>C	p.(Cys436Arg)	S1	increased potency	decreased potency	reduced	NA	NA	NA	NA	decreased	NA	LoF	Swanger SA, Chen W, Wells G, Burger PB, Tankovic A, Bhattacharya S, Strong KL, Hu C, Kusumoto H, Zhang J, Adams DR, Millichap JJ, Petrovski S, Traynelis SF, Yuan H.: Mechanistic Insight into NMDA Receptor Dysregulation by Rare Variants in the GluN2A and GluN2B Agonist Binding Domains. Am J Hum Genet 2016, 99(6):1261-1280.
c.1447G>A	p.(Gly483Arg)	S1	decreased potency	decreased potency	reduced	NA	NA	NA	NA	NA	NA	LoF	Addis L, Virdee JK, Vidler LR, Collier DA, Pal DK, Ursu D.: Epilepsy-associated GRIN2A mutations reduce NMDA receptor trafficking and agonist potency – molecular profiling and functional rescue. Sci Rep 2017, 7(1):66.
c.1447G>A	p.(Gly483Arg)	S1	decreased potency	no effect	reduced	NA	NA	NA	increased	decreased	NA	LoF	Swanger SA, Chen W, Wells G, Burger PB, Tankovic A, Bhattacharya S, Strong KL, Hu C, Kusumoto H, Zhang J, Adams DR, Millichap JJ, Petrovski S, Traynelis SF, Yuan H.: Mechanistic Insight into NMDA Receptor Dysregulation by Rare Variants in the GluN2A and GluN2B Agonist Binding Domains. Am J Hum Genet 2016, 99(6):1261-1280.
c.1510C>T	p.(Arg504Trp)	S1	no effect	no effect	NA	NA	NA	NA	increased	decreased (not significant)	NA	LoF	Swanger SA, Chen W, Wells G, Burger PB, Tankovic A, Bhattacharya S, Strong KL, Hu C, Kusumoto H, Zhang J, Adams DR, Millichap JJ, Petrovski S, Traynelis SF, Yuan H.: Mechanistic Insight into NMDA Receptor Dysregulation by Rare Variants in the GluN2A and GluN2B Agonist Binding Domains. Am J Hum Genet 2016, 99(6):1261-1280.
c.1553G>A	p.(Arg518His)	S1	no response	no response	reduced	NA	NA	NA	NA	decreased	NA	LoF	Swanger SA, Chen W, Wells G, Burger PB, Tankovic A, Bhattacharya S, Strong KL, Hu C, Kusumoto H, Zhang J, Adams DR, Millichap JJ, Petrovski S, Traynelis SF, Yuan H.: Mechanistic Insight into NMDA Receptor Dysregulation by Rare Variants in the GluN2A and GluN2B Agonist Binding Domains. Am J Hum Genet 2016, 99(6):1261-1280.
c.1592C>T	p.(Thr531Met)	S1	no response	no response	reduced	NA	NA	NA	increased	no current	NA	LoF	Swanger SA, Chen W, Wells G, Burger PB, Tankovic A, Bhattacharya S, Strong KL, Hu C, Kusumoto H, Zhang J, Adams DR, Millichap JJ, Petrovski S, Traynelis SF, Yuan H.: Mechanistic Insight into NMDA Receptor Dysregulation by Rare Variants in the GluN2A and GluN2B Agonist Binding Domains. Am J Hum Genet 2016, 99(6):1261-1280.
c.1642G>A	p.(Ala548Thr)	Linker	decreased potency	decreased potency	no effect	NA	NA	NA	NA	reduced	NA	LoF	Ogden KK, Chen W, Swanger SA, McDaniel MJ, Fan LZ, Hu C, et al. (2017) Molecular Mechanism of Disease-Associated Mutations in the Pre-M1 Helix of NMDA Receptors and Potential Rescue Pharmacology. PLoS Genet 13(1)
c.1655C>G	p.(Pro552Arg)	Linker	increased potency	increased potency	NA	NA	NA	NA	increased	decreased	NA	GoF	Ogden KK, Chen W, Swanger SA, McDaniel MJ, Fan LZ, Hu C, et al. (2017) Molecular Mechanism of Disease-Associated Mutations in the Pre-M1 Helix of NMDA Receptors and Potential Rescue Pharmacology. PLoS Genet 13(1)
c.1845C>A	p.(Asn615Lys)	M2	no effect	no effect	NA	eliminate block	NA	NA	NA	NA	decreased permeability	GoF	Endele S, Rosenberger G, Geider K, Popp B, Tamer C, Stefanova I, Milh M, Kortüm F, Fritsch A, Pientka FK, Hellenbroich Y, Kalscheuer VM, Kohlhase J, Moog U, Rappold G, Rauch A, Ropers HH, von Spiczak S, Tönnies H, Villeneuve N, Villard L, Zabel B, Zenker M, Laube B, Reis A, Wieczorek D, Van Maldergem L, Kutsche K.: Mutations in GRIN2A and GRIN2B encoding regulatory subunits of NMDA receptors cause variable neurodevelopmental phenotypes. Nat Genet 2010, 42(11):1021-6.
c.1954T>G	p.(Phe652Val)	M3	NA	NA	NA	NA	NA	NA	decreased	NA	NA	GoF	Lesca G, Rudolf G, Bruneau N, Lozovaya N, Labalme A, Boutry-Kryza N, Salmi M, Tsintsadze T, Addis L, Motte J, Wright S, Tsintsadze V, Michel A, Doummar D, Lascelles K, Strug L, Waters P, de Bellescize J, Vrielynck P, de Saint Martin A, Ville D, Ryvlin P, Arzimanoglou A, Hirsch E, Vincent A, Pal D, Burnashev N, Sanlaville D, Szepetowski P.: GRIN2A mutations in acquired epileptic aphasia and related childhood focal epilepsies and encephalopathies with speech and language dysfunction. Nat Genet 2013, 45(9):1061-6.
c.2054T>C	p.(Val685Gly)	S2	decreased potency	no effect	reduced	NA	NA	NA	decreased (not significant)	decreased	NA	LoF	Swanger SA, Chen W, Wells G, Burger PB, Tankovic A, Bhattacharya S, Strong KL, Hu C, Kusumoto H, Zhang J, Adams DR, Millichap JJ, Petrovski S, Traynelis SF, Yuan H.: Mechanistic Insight into NMDA Receptor Dysregulation by Rare Variants in the GluN2A and GluN2B Agonist Binding Domains. Am J Hum Genet 2016, 99(6):1261-1280.
c.2081T>C	p.(Ile694Thr)	S2	decreased potency	no effect	reduced	NA	NA	NA	no effect	decreased	NA	LoF	Swanger SA, Chen W, Wells G, Burger PB, Tankovic A, Bhattacharya S, Strong KL, Hu C, Kusumoto H, Zhang J, Adams DR, Millichap JJ, Petrovski S, Traynelis SF, Yuan H.: Mechanistic Insight into NMDA Receptor Dysregulation by Rare Variants in the GluN2A and GluN2B Agonist Binding Domains. Am J Hum Genet 2016, 99(6):1261-1280.
c.2095C>T	p.(Pro699Ser)	S2	decreased potency	no effect	reduced	NA	NA	NA	NA	decreased (not significant)	NA	LoF	Swanger SA, Chen W, Wells G, Burger PB, Tankovic A, Bhattacharya S, Strong KL, Hu C, Kusumoto H, Zhang J, Adams DR, Millichap JJ, Petrovski S, Traynelis SF, Yuan H.: Mechanistic Insight into NMDA Receptor Dysregulation by Rare Variants in the GluN2A and GluN2B Agonist Binding Domains. Am J Hum Genet 2016, 99(6):1261-1280.
c.2113A>G	p.(Met705Val)	S2	decreased potency	decreased potency	reduced (not significant)	NA	NA	NA	NA	NA	NA	LoF	Addis L, Virdee JK, Vidler LR, Collier DA, Pal DK, Ursu D.: Epilepsy-associated GRIN2A mutations reduce NMDA receptor trafficking and agonist potency – molecular profiling and functional rescue. Sci Rep 2017, 7(1):66.

c.2113A>G	p.(Met705Val)	S2	decreased potency	no effect	reduced	NA	NA	NA	increased (not significant)	decreased (not significant)	NA	LoF	Swanger SA, Chen W, Wells G, Burger PB, Tankovic A, Bhattacharya S, Strong KL, Hu C, Kusumoto H, Zhang J, Adams DR, Millichap JJ, Petrovski S, Traynelis SF, Yuan H.: Mechanistic Insight into NMDA Receptor Dysregulation by Rare Variants in the GluN2A and GluN2B Agonist Binding Domains. Am J Hum Genet 2016, 99(6):1261-1280.
c.2146G>A	p.(Ala716Thr)	S2	decreased potency	no effect	reduced	NA	NA	NA	decreased (not significant)	decreased (not significant)	NA	LoF	Swanger SA, Chen W, Wells G, Burger PB, Tankovic A, Bhattacharya S, Strong KL, Hu C, Kusumoto H, Zhang J, Adams DR, Millichap JJ, Petrovski S, Traynelis SF, Yuan H.: Mechanistic Insight into NMDA Receptor Dysregulation by Rare Variants in the GluN2A and GluN2B Agonist Binding Domains. Am J Hum Genet 2016, 99(6):1261-1280.
c.2179G>A	p.(p.Ala727Thr)	S2	decreased potency	no effect	reduced	NA	NA	NA	no effect	decreased (not significant)	NA	LoF	Swanger SA, Chen W, Wells G, Burger PB, Tankovic A, Bhattacharya S, Strong KL, Hu C, Kusumoto H, Zhang J, Adams DR, Millichap JJ, Petrovski S, Traynelis SF, Yuan H.: Mechanistic Insight into NMDA Receptor Dysregulation by Rare Variants in the GluN2A and GluN2B Agonist Binding Domains. Am J Hum Genet 2016, 99(6):1261-1280.
c.2191G>A	p.(Asp731Asn)	S2	no response	no response	reduced	NA	NA	NA	NA	NA	NA	LoF	Addis L, Virdee JK, Vidler LR, Collier DA, Pal DK, Ursu D.: Epilepsy-associated GRIN2A mutations reduce NMDA receptor trafficking and agonist potency – molecular profiling and functional rescue. Sci Rep 2017, 7(1):66.
c.2191G>A	p.(Asp731Asn)	S2	decreased potency	no effect	reduced	NA	NA	NA	NA	decreased	decreased permeability	LoF	Swanger SA, Chen W, Wells G, Burger PB, Tankovic A, Bhattacharya S, Strong KL, Hu C, Kusumoto H, Zhang J, Adams DR, Millichap JJ, Petrovski S, Traynelis SF, Yuan H.: Mechanistic Insight into NMDA Receptor Dysregulation by Rare Variants in the GluN2A and GluN2B Agonist Binding Domains. Am J Hum Genet 2016, 99(6):1261-1280.
c.2200G>C	p.(Val734Leu)	S2	decreased potency	no effect	no effect	NA	NA	NA	decreased	decreased (not significant)	NA	LoF	Swanger SA, Chen W, Wells G, Burger PB, Tankovic A, Bhattacharya S, Strong KL, Hu C, Kusumoto H, Zhang J, Adams DR, Millichap JJ, Petrovski S, Traynelis SF, Yuan H.: Mechanistic Insight into NMDA Receptor Dysregulation by Rare Variants in the GluN2A and GluN2B Agonist Binding Domains. Am J Hum Genet 2016, 99(6):1261-1280.
c.2314A>G	p.(Lys772Glu)	S2	decreased potency	no effect	reduced	NA	NA	NA	no effect	decreased	NA	LoF	Swanger SA, Chen W, Wells G, Burger PB, Tankovic A, Bhattacharya S, Strong KL, Hu C, Kusumoto H, Zhang J, Adams DR, Millichap JJ, Petrovski S, Traynelis SF, Yuan H.: Mechanistic Insight into NMDA Receptor Dysregulation by Rare Variants in the GluN2A and GluN2B Agonist Binding Domains. Am J Hum Genet 2016, 99(6):1261-1280.
c.2434C>A	p.(Leu812Met)	Linker	increased potency	increased potency	NA	increased block	reduced sensitivity	reduced sensitivity	NA	NA	no effect	GoF	Yuan H, Hansen KB, Zhang J, Pierson TM, Markello TC, Fajardo KV, Holloman CM, Golas G, Adams DR, Boerkoel CF, Gahl WA, Traynelis SF.: Functional analysis of a de novo GRIN2A missense mutation associated with early-onset epileptic encephalopathy. Nat Commun 2014, 5:3251. Pierson TM, Yuan H, Marsh ED, Fuentes-Fajardo K, Adams DR, Markello T, Golas G, Simeonov DR, Holloman C, Tankovic A, Karamchandani MM, Schreiber JM, Mullikin JC; PhD for the NISC Comparative Sequencing Program, Tifft CJ, Toro C, Boerkoel CF, Traynelis SF, Gahl WA.: GRIN2A mutation and early-onset epileptic encephalopathy: personalized therapy with memantine. Ann Clin Transl Neurol 2014, 1(3):190-198.
c.2450T>C	p.(Met817Thr)	M4	increased potency	increased potency	NA	reduced sensitivity	reduced sensitivity	reduced sensitivity	NA	NA	NA	GoF	Chen W, Tankovic A, Burger PB, Kusumoto H, Traynelis SF, Yuan H.: Functional Evaluation of a De Novo GRIN2A Mutation Identified in a Patient with Profound Global Developmental Delay and Refractory Epilepsy. Mol Pharmacol 2017, 91(4):317-330.
