# Supplementary material for: GRIN2A-related disorders: genotype and functional consequence predict phenotype
Source: Brain. 2018 Dec 12;142(1):80–92. doi: 10.1093/brain/awy304 (PMC6308310; doi:10.1093/brain/awy304)
Supplement: Supplementary Data [file awy304_supp.zip › awy304-suppl_data/brain-2018-01122-File014.pdf]

#	DNA	PROTEIN	DOMAIN	ID/DD	SCORE
001	c.236C>G	p.(Pro79Arg)	ATD	mild	1
002	c.236C>G	p.(Pro79Arg)	ATD	no	0
003	c.236C>G	p.(Pro79Arg)	ATD	no	0
004	c.236C>G	p.(Pro79Arg)	ATD	no	0
005	c.236C>G	p.(Pro79Arg)	ATD	no	0
006	c.236C>G	p.(Pro79Arg)	ATD	NA	NA
007	c.551T>G	p.(Ile184Ser)	ATD	mild	1
008	c.551T>G	p.(Ile184Ser)	ATD	NA	NA
009	c.691T>C	p.(Cys231Arg)	ATD	unspecified	NA
010	c.692G>A	p.(Cys231Tyr)	ATD	mild	1
011	c.692G>A	p.(Cys231Tyr)	ATD	NA	NA
012	c.692G>A	p.(Cys231Tyr)	ATD	NA	NA
013	c.692G>A	p.(Cys231Tyr)	ATD	NA	NA
014	c.1232T>A	p.(Leu411Gln)	S1	severe	3
015	c.1306T>C	p.(Cys436Arg)	S1	no	0
016	c.1306T>C	p.(Cys436Arg)	S1	NA	NA
017	c.1447G>A	p.(Gly483Arg)	S1	unspecified	NA
018	c.1447G>A	p.(Gly483Arg)	S1	unspecified	NA
019	c.1492G>A	p.(Gly498Ser)	S1	mild	1
020	c.1552C>T	p.(Arg518Cys)	S1	NA	NA
021	c.1552C>T	p.(Arg518Cys)	S1	unspecified	NA
022	c.1553G>A	p.(Arg518His)	S1	unspecified	NA
023	c.1553G>A	p.(Arg518His)	S1	no	0
024	c.1553G>A	p.(Arg518His)	S1	no	0
025	c.1553G>A	p.(Arg518His)	S1	no	0
026	c.1553G>A	p.(Arg518His)	S1	no	0
027	c.1592C>T	p.(Thr531Met)	S1	moderate	2
028	c.1592C>T	p.(Thr531Met)	S1	severe	3
029	c.1592C>T	p.(Thr531Met)	S1	no	0
030	c.1592C>T	p.(Thr531Met)	S1	no	0
031	c.1592C>T	p.(Thr531Met)	S1	mild	1
032	c.1592C>T	p.(Thr531Met)	S1	no	0
033	c.1592C>T	p.(Thr531Met)	S1	mild	1
034	c.1595G>T	p.(Gly532Val)	S1	unspecified	NA
035	c.1642G>A	p.(Ala548Thr)	Linker	moderate	2
036	c.1642G>C	p.(Ala548Thr)	Linker	unspecified	NA
037	c.1655C>G	p.(Pro552Arg)	Linker	profound	4
038	c.1655C>G	p.(Pro552Arg)	Linker	profound	4
039	c.1832T>A	p.(Leu611Gln)	M2	unspecified	NA
040	c.1841A>G	p.(Asn614Ser)	M2	profound	4
041	c.1841A>G	p.(Asn614Ser)	M2	NA	NA
042	c.1841A>G	p.(Asn614Ser)	M2	profound	4
043	c.1841A>G	p.(Asn614Ser)	M2	profound	4
044	c.1845C>A	p.(Asn615Lys)	M2	profound	4
045	c.1845C>A	p.(Asn615Lys)	M2	profound	4

046	c.1903G>A	p.(Ala635Thr)	M3	profound	4
047	c.1930A>G	p.(Ser644Gly)	M3	NA	NA
048	c.1936A>G	p.(Thr646Ala)	M3	profound	4
049	c.1936A>G	p.(Thr646Ala)	M3	NA	NA
050	c.1943A>G	p.(Asn648Ser)	M3	NA	NA
051	c.1943A>G	p.(Asn648Ser)	M3	NA	NA
052	c.1943A>G	p.(Asn648Ser)	M3	unspecified	NA
053	c.1945C>G	p.(Leu649Val)	M3	profound	4
054	c.1945C>G	p.(Leu649Val)	M3	profound	4
055	c.1946_1947delinsCT	p.(Leu649Pro)	M3	profound	4
056	c.1954T>G	p.(Phe652Val)	M3	unspecified	NA
057	c.1957A>G	p.(Met653Val)	M3	unspecified	NA
058	c.1959G>A	p.(Met653Ile)	M3	severe	3
059	c.1961T>C	p.(Ile654Thr)	M3	profound	4
060	c.2050A>G	p.(Thr684Ala)	S2	unspecified	NA
061	c.2063G>C	p.(Gly688Ala)	S2	no	0
062	c.2081T>C	p.(Ile694Thr)	S2	no	0
063	c.2084G>A	p.(Arg695Gln)	S2	moderate	2
064	c.2084G>A	p.(Arg695Gln)	S2	unspecified	NA
065	c.2095C>T	p.(Pro699Ser)	S2	no	0
066	c.2095C>T	p.(Pro699Ser)	S2	NA	NA
067	c.2113A>G	p.(Met705Val)	S2	no	0
068	c.2113A>G	p.(Met705Val)	S2	no	0
069	c.2113A>G	p.(Met705Val)	S2	no	0
070	c.2138T>G	p.(Val713Gly)	S2	unspecified	NA
071	c.2146G>A	p.(Ala716Thr)	S2	NA	NA
072	c.2146G>A	p.(Ala716Thr)	S2	NA	NA
073	c.2146G>A	p.(Ala716Thr)	S2	NA	NA
074	c.2146G>A	p.(Ala716Thr)	S2	NA	NA
075	c.2146G>A	p.(Ala716Thr)	S2	NA	NA
076	c.2146G>A	p.(Ala716Thr)	S2	NA	NA
077	c.2146G>A	p.(Ala716Thr)	S2	NA	NA
078	c.2146G>A	p.(Ala716Thr)	S2	NA	NA
079	c.2146G>A	p.(Ala716Thr)	S2	NA	NA
080	c.2146G>A	p.(Ala716Thr)	S2	NA	NA
081	c.2146G>A	p.(Ala716Thr)	S2	NA	NA
082	c.2146G>A	p.(Ala716Thr)	S2	no	0
083	c.2191G>A	p.(Asp731Asn)	S2	severe	3
084	c.2191G>A	p.(Asp731Asn)	S2	NA	NA
085	c.2191G>A	p.(Asp731Asn)	S2	NA	NA
086	c.2191G>A	p.(Asp731Asn)	S2	NA	NA
087	c.2191G>A	p.(Asp731Asn)	S2	NA	NA
088	c.2191G>A	p.(Asp731Asn)	S2	unspecified	NA
089	c.2197G>A	p.(Ala733Thr)	S2	NA	NA
090	c.2278G>A	p.(Gly760Ser)	S2	unspecified	NA
091	c.2326G>T	p.(Asp776Tyr)	S2	no	0

092	c.2326G>T	p.(Asp776Tyr)	S2	no	0
093	c.2326G>T	p.(Asp776Tyr)	S2	no	0
094	c.2427C>A	p.(Ser809Arg)	Linker	unspecified	NA
095	c.2434C>A	p.(Leu812Met)	Linker	profound	4
096	c.2449A>G	p.(Met817Val)	M4	profound	4
097	c.2449A>G	p.(Met817Val)	M4	NA	NA
098	c.2449A>G	p.(Met817Val)	M4	NA	NA
099	c.2450T>C	p.(Met817Thr)	M4	mild	1
100	c.2450T>C	p.(Met817Thr)	M4	NA	NA
101	c.2450T>G	p.(Met817Thr)	M4	unspecified	NA
102	c.2453C>A	p.Ala818Glu	M4	moderate	2
103	c.2T>C	p.(Met1?)	NA	mild	1
104	c.2T>C	p.(Met1?)	NA	mild	1
105	c.2T>C	p.(Met1?)	NA	no	0
106	c.90dupT	p.(Pro31Serfs*107)	NA	NA	NA
107	c.90dupT	p.(Pro31Serfs*107)	NA	NA	NA
108	c.90dupT	p.(Pro31Serfs*107)	NA	NA	NA
109	c.90dupT	p.(Pro31Serfs*107)	NA	NA	NA
110	c.165G>A	p.(Trp55*)	NA	unspecified	NA
111	c.172G>T	p.(Glu58*)	NA	moderate	2
112	c.172G>T	p.(Glu58*)	NA	NA	NA
113	c.176_179dupAGGC	p.(Ala61Glyfs*78)	NA	mild	1
114	c.415-2A>G	NA	NA	mild	1
115	c.445_448delGCGTins69	p.(Ala149Serfs*8)	NA	unspecified	NA
116	c.487C>T	p.(Gln163*)	NA	unspecified	NA
117	c.487C>T	p.(Gln163*)	NA	unspecified	NA
118	c.500G>A	p.(Trp167*)	NA	mild	1
119	c.594G>A	p.(Trp198*)	NA	mild	1
120	c.594G>A	p.(Trp198*)	NA	mild	1
121	c.594G>A	p.(Trp198*)	NA	mild	1
122	c.594G>A	p.(Trp198*)	NA	no	0
123	c.594G>A	p.(Trp198*)	NA	no	0
124	c.594G>A	p.(Trp198*)	NA	no	0
125	c.594G>A	p.(Trp198*)	NA	no	0
126	c.594G>A	p.(Trp198*)	NA	unspecified	NA
127	c.594G>A	p.(Trp198*)	NA	no	0
128	c.594G>A	p.(Trp198*)	NA	no	0
129	c.627delC	p.(Phe210Leufs*10)	NA	NA	NA
130	c.627delC	p.(Phe210Leufs*10)	NA	NA	NA
131	c.652C>T	p.(Gln218*)	NA	mild	1
132	c.652C>T	p.(Gln218*)	NA	no	0
133	c.652C>T	p.(Gln218*)	NA	no	0
134	c.1001T>A	p.(Leu334*)	NA	no	0
135	c.1001T>A	p.(Leu334*)	NA	no	0
136	c.1001T>A	p.(Leu334*)	NA	no	0
137	c.1007+1G>A	NA	NA	no	0

138	c.1007+1G>A	NA	NA	no	0
139	c.1007+1G>A	NA	NA	no	0
140	c.1007+1G>A	NA	NA	no	0
141	c.1007+1G>A	NA	NA	no	0
142	c.1007+1G>A	NA	NA	mild	1
143	c.1007+1G>A	NA	NA	no	0
144	c.1007+1G>A	NA	NA	moderate	2
145	c.1007+1G>A	NA	NA	mild	1
146	c.1007+1G>A	NA	NA	unspecified	NA
147	c.1007+1G>A	NA	NA	NA	NA
148	c.1007+1G>A	NA	NA	NA	NA
149	c.1007+1G>A	NA	NA	NA	NA
150	c.1007+1G>A	NA	NA	unspecified	NA
151	c.1007+1G>A	NA	NA	NA	NA
152	c.1007+1G>A	NA	NA	NA	NA
153	c.1007+1G>T	NA	NA	mild	1
154	c.1007+1G>T	NA	NA	NA	NA
155	c.1007+1G>A	NA	NA	moderate	2
156	c.1007+1G>A	NA	NA	unspecified	NA
157	c.1007+1G>A	NA	NA	unspecified	NA
158	c.1007+1G>A	NA	NA	NA	NA
159	c.1007+1G>A	NA	NA	NA	NA
160	c.1007+1G>A	NA	NA	NA	NA
161	c.1007+1G>A	NA	NA	mild	1
162	c.1036A>T	p.(Lys346*)	NA	mild	1
163	c.2008-1G>T	NA	NA	unspecified	NA
164	c.1123-1G>T	NA	NA	mild	1
165	c.1123-1G>T	NA	NA	NA	NA
166	c.1123-2A>G	NA	NA	no	0
167	c.1123-2A>G	NA	NA	no	0
168	c.1123-2A>G	NA	NA	NA	NA
169	c.1123-2A>G	NA	NA	NA	NA
170	c.1123-2A>G	NA	NA	mild	1
171	c.1123-2A>G	NA	NA	NA	NA
172	c.1123-2A>G	NA	NA	NA	NA
173	c.1344_1345insCCTAG	p.(Gly449Profs*4)	NA	NA	NA
174	c.1362delA	p.(Lys454Asnfs*11)	NA	no	0
175	c.1497+1G>C	NA	NA	NA	NA
176	c.1585delG	p.(Val529Trpfs*22)	NA	mild	1
177	c.1585delG	p.(Val529Trpfs*22)	NA	no	0
178	c.1585delG	p.(Val529Trpfs*22)	NA	no	0
179	c.1586delT	p.(Val529Glyfs*22)	NA	moderate	2
180	c.1613C>G	p.(Ser538*)	NA	moderate	2
181	c.1651+1del	NA	NA	no	0
182	c.1686del	p.(Phe562Leufs*2)	NA	mild	1
183	c.1692delG	p.(Met564Ilefs*8)	NA	NA	NA

184	c.1782_1783insTT	p.(His595Serfs*60)	NA	NA	NA
185	c.1783_1784delCA	p.(His595Trpfs*20)	NA	NA	NA
186	c.1818G>A	p.(Trp606*)	NA	mild	1
187	c.2007+1G>A	NA	NA	no	0
188	c.2007+1G>A	NA	NA	NA	NA
189	c.2007+2dup	NA	NA	no	0
190	c.2008-1G>T	NA	NA	unspecified	NA
191	c.2041C>T	p.(Arg681*)	NA	no	0
192	c.2041C>T	p.(Arg681*)	NA	no	0
193	c.2041C>T	p.(Arg681*)	NA	NA	NA
194	c.2041C>T	p.(Arg681*)	NA	NA	NA
195	c.2041C>T	p.(Arg681*)	NA	no	0
196	c.2041C>T	p.(Arg681*)	NA	severe	3
197	c.2041C>T	p.(Arg681*)	NA	no	0
198	c.2041C>T	p.(Arg681*)	NA	no	0
199	c.2041C>T	p.(Arg681*)	NA	NA	NA
200	c.2140delG	p.(Glu714Argfs*7)	NA	NA	NA
201	c.2253dupG	p.(Ser752Glufs*34)	NA	mild	1
202	c.2334_2338delCTTGC	p.(Leu779Serfs*5)	NA	no	0
203	c.2341_2343delinsAT	p.(Gln781Ilefs*27)	NA	mild	1
204	c.2407G>T	p.(Glu803*)	NA	moderate	2
205	c.2408del	p.(Glu803Glyfs*5)	NA	moderate	2
206	Del Exon 1-3	NA	NA	NA	0
207	Del Exon 1-3	NA	NA	NA	1
208	Del Exon 1-3	NA	NA	NA	2
209	Del Exon 1-3	NA	NA	no	0
210	Del Exon 1-3	NA	NA	mild	1
211	Del Exon 1-3	NA	NA	no	0
212	Del Exon 1-3	NA	NA	NA	NA
213	Del Exon 1-3	NA	NA	moderate	2
214	Del Exon 1-3	NA	NA	moderate	2
215	Del Exon 1-3	NA	NA	moderate	2
216	Del Exon 1-3	NA	NA	profound	4
217	Del Exon 1-3	NA	NA	unspecified	NA
218	Del Exon 1-3	NA	NA	unspecified	NA
219	Del Exon 1-14	NA	NA	NA	1
220	Del Exon 1-14	NA	NA	unspecified	NA
221	Del Exon 4	NA	NA	mild	1
222	Del Exon 4	NA	NA	no	0
223	Del Exon 4	NA	NA	no	0
224	Del Exon 4	NA	NA	no	0
225	Del Exon 4	NA	NA	mild	1
226	Del Exon 4-5	NA	NA	no	0
227	Del Exon 4-5	NA	NA	no	0
228	Del Exon 4-6	NA	NA	unspecified	NA
229	Del Exon 4-14	NA	NA	unspecified	NA

230	Del Exon 4-14	NA	NA	no	0
231	Del Exon 4-14	NA	NA	no	0
232	Del Exon 6-7	NA	NA	mild	1
233	Del Exon 6-7	NA	NA	moderate	2
234	Del Exon 6-7	NA	NA	mild	1
235	Del Exon 6-11	NA	NA	severe	3
236	Del Exon 11	NA	NA	no	0
237	Del Exon 11	NA	NA	mild	1
238	Del Exon 11	NA	NA	moderate	2
239	Del Exon 11	NA	NA	NA	1
240	Del Exon 12-14	NA	NA	NA	1
241	Dup Exon 3-4	NA	NA	mild	1
242	Dup Exon 4-5	NA	NA	severe	3
243	t(16;17)(p13.2;q11.2)	NA	NA	severe	3
244	t(16;17)(p13.2;q11.2)	NA	NA	no	0
245	t(16;17)(p13.2;q11.2)	NA	NA	no	0
246	t(16;17)(p13.2;q11.2)	NA	NA	moderate	2
247	arr[GRCh37] 16p13.2 (9781694_9867779)x3, 16p13.2(10001541_10230382)x3	NA	NA	unspecified	NA
