# Supplementary material for: GRIN2A-related disorders: genotype and functional consequence predict phenotype
Source: Brain. 2018 Dec 12;142(1):80–92. doi: 10.1093/brain/awy304 (PMC6308310; doi:10.1093/brain/awy304)
Supplement: Supplementary Data [file awy304_supp.zip › awy304-suppl_data/brain-2018-01122-File015.pdf]

Start	End	Domain	
1	22	signal peptide	
23	404	ATD	
405	539	S1 ABD	
540	555	Linker	
556	576	M1	TMD
598	622	M2	TMD
634	654	M3	TMD
655	660	Linker	
661	801	S2 ABD	
802	816	Linker	
817	837	M4	TMD
838	1469	CTD	
