# Supplementary material for: GRIN2A-related disorders: genotype and functional consequence predict phenotype
Source: Brain. 2018 Dec 12;142(1):80–92. doi: 10.1093/brain/awy304 (PMC6308310; doi:10.1093/brain/awy304)
Supplement: Supplementary Data [file awy304_supp.zip › awy304-suppl_data/brain-2018-01122-File017.pdf]

Full blots for Figure 6A

GluN2A N-terminus

Grin2a genotype

+ve control | +/+$^{50\%}$ | +/+ | +/- | -/- | +/+ | +/- | -/- | +/+ | +/- | -/- | +/+ | +/- | -/-

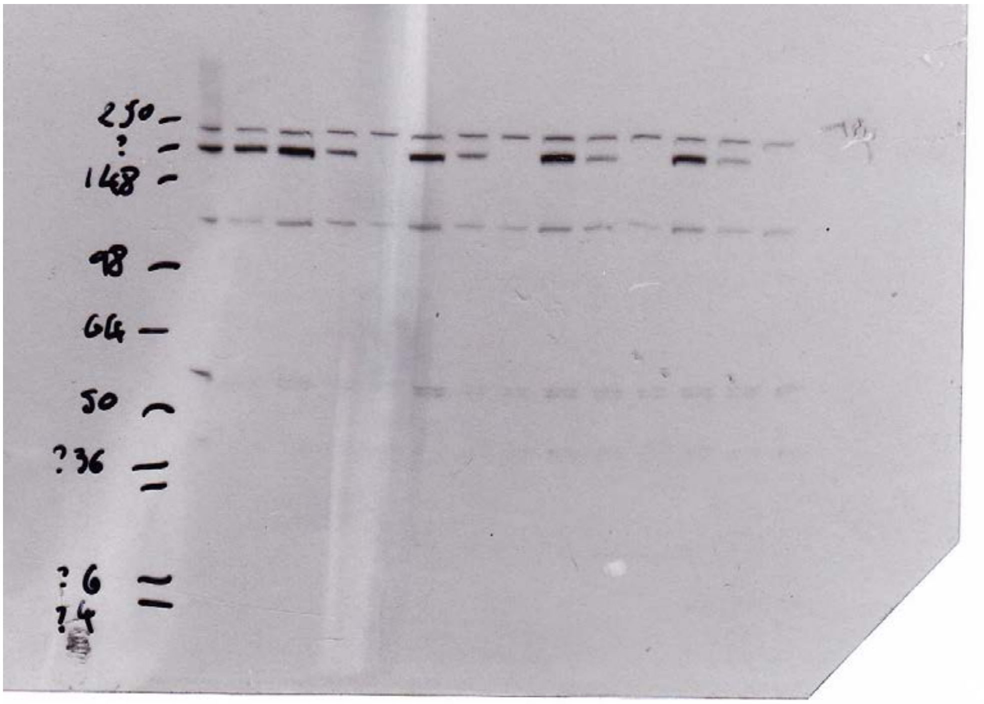

β actin

Grin2a genotype

+ve control | +/+$^{50\%}$ | +/+ | +/- | -/- | +/+ | +/- | -/- | +/+ | +/- | -/- | +/+ | +/- | -/-

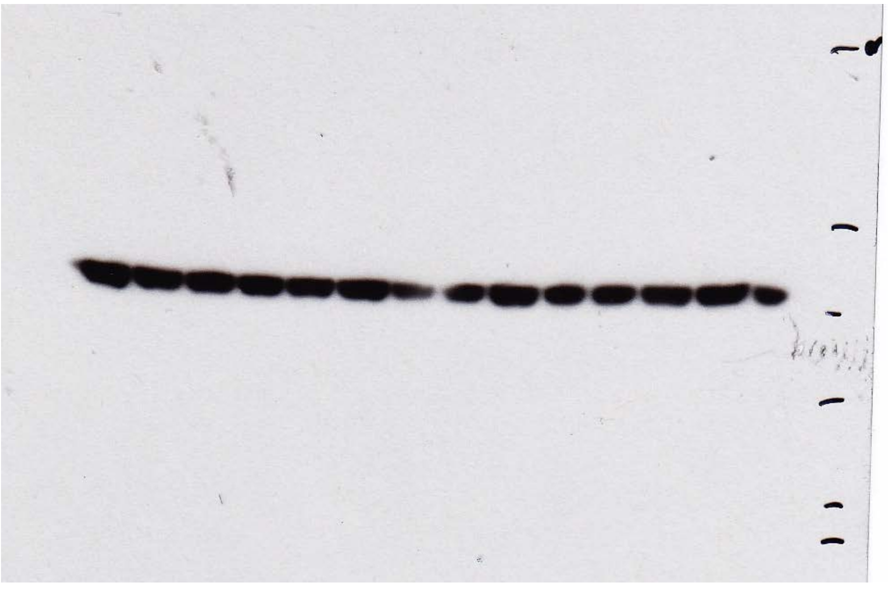

+/+$^{50\%}$: WT sample diluted 2 fold

Full blots for Figure 6B

GluN2B C-terminus
(blot cut in half, lower portion used for β actin)

Grin2a genotype

+/+ +/- -/- +/+ +/- -/- +/+ +/- -/- +/+ +/- -/- +/+ +/- -/- +/+ -/- +/- -/-

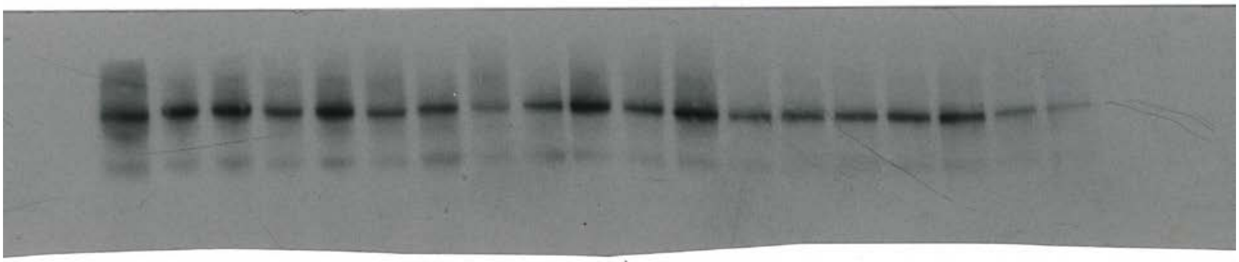

β actin
(lower portion of above blot)

Grin2a genotype

+/+ +/- -/- +/+ +/- -/- +/+ +/- -/- +/+ +/- -/- +/+ +/- -/- +/+ -/- +/- -/-
