# Supplementary material for: GRIN2A-related disorders: genotype and functional consequence predict phenotype
Source: Brain. 2018 Dec 12;142(1):80–92. doi: 10.1093/brain/awy304 (PMC6308310; doi:10.1093/brain/awy304)
Supplement: Supplementary Data [file awy304_supp.zip › awy304-suppl_data/brain-2018-01122-File019.pdf]

Supplementary figure 1: Distribution of phenotypes

Individuals with *GRIN2A*-related disorders display a broad range of phenotype severity and expressivity with respect to **(A)** hypotonia, **(B)** movement disorders and **(C)** MRI findings.

Supplementary figure 2: ***Variance of ID/DD phenotype in individuals with the same genetic variant***

The mean variance of ID/DD phenotypes per variant was 0.65 (±0.64 SD). Permuting family labels 10,000 times revealed a lower value than the mean variance in 15 of 10,000 permutations (empirical *P* -value 0.0016).

Supplementary figure 3: Confirmation of GluN2A expression at DIV15-16

Evidence for the developmental increase in the expression of GluN2A by DIV15 in $Grin2a^{+/+}$ neurons. **(A)** Western blot indicating the increase in GluN2A protein at DIV15 compared to DIV7. **(B)** *Grin2a* mRNA levels are significantly increased (P<0.0001, t-test) at DIV15 (n=8) compared to DIV7 (n=8). Similarly GluN2A protein is significantly increased (P<0.0118, t-test) at DIV15 (n=6) compared to DIV7 (n=6). **(C)** At DIV15-16 there is significantly less potentiation by spermine (100 µM) of NMDA (150 µM) evoked currents as would be expected for neurons where GluN2A expression was present (P=0.011, t-test). DIV7-8: n=25 cells; DIV15-16: n=25 cells, 8 animals.

Supplementary figure 4 : Western Blots

Original full-length Western blots used for Figures 6A and B of this manuscript

Supplementary table 1: Phenotype questionnaire

Supplementary table 2: All Individuals

Supplementary table 3: Functional characterisation

Supplementary table 4: ID/DD score

Supplementary table 5: Protein domains
