# Supplementary material for: GRIN2A-related disorders: genotype and functional consequence predict phenotype
Source: Brain. 2018 Dec 12;142(1):80–92. doi: 10.1093/brain/awy304 (PMC6308310; doi:10.1093/brain/awy304)
Supplement: Supplementary Data [file awy304_supp.zip › awy304-suppl_data/brain-2018-01122-File020.pdf]

GRIN2A study group

Paul B. Augustijn[1], Patrick Van Bogaert[2], Helene Bourry[3], Peter Burfeind[4], Yoyo Chu[5], Brian Chung[5], Diane Doummar[6], Patrick Edery[38,39,40], Aviva Fattal-Valevski[7,8], Mélanie Fradin[9], Marion Gerard[10], Christa de Geus[5], Boudewijn Gunning[11], Danielle Hasaerts[12], Ingo Helbig[13], Katherine L. Helbig[13], Rami Jamra[1], Mélanie Jennesson Lyver[14], Jolien S. Klein Wassink-Ruiter[5], David A. Koolen[15], Damien Lederer[16], Roelineke J. Lunsing[4], Mikaël Mathot[17],Hélène Maurey[18], Shay Menascu[8,19], Anne Michel[20], Ghayda Mirzaa[21,22], Diana Mitter[1], Hiltrud Muhle[23], Rikke S. Møller[24,25], Caroline Nava[26], Margaret O`Brien[13], Evelyn van Pinxteren-Nagler[27], Anne van Riesen[28], Christelle Rougeot[29], Damien Sanlaville[20,21,22,], Jolanda H. Schieving[30], Steffen Syrbe[31], Hermine E. Veenstra-Knol[5], Nienke Verbeek[13], Dorothée Ville[32], Yvonne J. Vos[5], Pascal Vrielynck[33], Sabrina Wagner[29], Sarah Weckhuysen[34,35,36], Marjolein H. Willemsen[15,37]

1 Department of Child Epileptology, Stichting Epilepsie Instellingen Nederland (S.E.I.N.), Heemstede, The Netherlands

2 Department of Pediatric Neurology, Angers University Hospital, Angers, France

3 Unité Pédiatrique Médicale, Centre Hospitalier Universitaire de Caen, Caen, France

4 Institute of Human Genetics, University Medical Centre Göttingen, Germany

5 Department of Pediatric and Adolescent Medicine, Queen Mary Hospital, University of Hong Kong, Hong Kong

6 Assistance Publique-Hôpitaux de Paris, Department of Pediatric Neurology, Hospital Armand Trousseau, Paris, France

7 Pediatric Neurology, Tel Aviv Sourasky Medical Center and Sackler Faculty of Medicine, Tel Aviv University, Tel Aviv, Israel

8 Sackler Faculty of Medicine, Tel-Aviv University, Tel-Aviv, Israel

9 Service de Génétique Clinique, CHU Rennes, Rennes, France

10 Génétique Clinique, Centre Hospitalier Universitaire de Caen, Caen, France

11 Department of Child Epileptology, Stichting Epilepsie Instellingen Nederland (S.E.I.N.), Zwolle, The Netherlands

12 Department of Child Neurology, Academic Children's Hospital, Vrije Universiteit Brussel, Brussels, Belgium

13 Division of Neurology, The Children's Hospital of Philadelphia, Philadelphia, PA, USA

14 Department of Pediatric Neurology, American Memorial Hospital, CHU Reims, Reims, France

15 Department of Human Genetics, Radboud University Medical Center, Nijmegen, the Netherlands

16 Centre de Génétique Humaine, Institut de Pathologie et de Génétique, Charleroi, Gosselies, Belgium

17 Neuropediatric unit, CHU UCL-Namur, Namur, Belgium

18 Department of Paediatric Neurology, Hôpital Bicêtre, Assistance publique des Hôpitaux de Paris, Le Kremlin-Bicêtre, France

19 Multiple Sclerosis Center, Sheba Medical Center, Tel Hashomer, Israel

20 UCB BioPharma, Braine L'Alleud, Belgium

21 Division of Genetic Medicine, Department of Pediatrics, University of Washington, Seattle, WA, USA

22 Center for Integrative Brain Research, Seattle Children's Research Institute, Seattle, WA, USA

23 Department of Neuropediatrics, Christian-Albrechts-University of Kiel, Kiel, Germany

24 Danish Epilepsy Centre, Dianalund, Denmark

25 University of Southern Denmark, Institute for Regional Health research, Odense, Denmark

26 Unité Fonctionnelle de Génomique du Développement, Centre de Génétique Moléculaire et Chromosomique, CHU Paris-GH La Pitié Salpêtrière-Charles Foix, Paris, France

27 Medical Centre Leeuwarden, Department of Paediatrics, Leeuwarden, The Netherlands

28 Department of Neuropediatrics, Charité Universitätsmedizin Berlin, Berlin, Germany

29 Pediatric Neurology Department, Lyon University Hospital, Bron, France

30 Department of Pediatric Neurology, University of Nijmegen, Radboud University Medical Centre, Nijmegen, The Netherlands

31 Department of General Paediatrics, Division of Child Neurology and Inherited Metabolic Diseases, Centre for Paediatrics and Adolescent Medicine, University Hospital Heidelberg, Heidelberg, Germany

32 Department of Pediatric Neurology and Reference Center for Rare Children Epilepsy and Tuberous Sclerosis, Hôpital Femme Mere Enfant, Centre Hospitalier Universitaire de Lyon, France

33 Department of Neurology, Centre Hospitalier Neurologique William Lennox, Ottignies, Belgium

34 Neurogenetics Group, Center for Molecular Neurology, VIB, Antwerp, Belgium

35 Laboratory of Neurogenetics, Institute Born-Bunge, University of Antwerp, Belgium

36 Division of Neurology, Antwerp University Hospital, Antwerp, Belgium

37 Department of Human Genetics, Maastricht University Medical Centre, Maastricht The Netherlands

38 Department of Genetics, Lyon University Hospitals, Lyon, France

39 Lyon Neuroscience Research Centre, CNRS UMR5292, INSERM U1028, Lyon, France

40 Claude Bernard Lyon I University, Lyon, France
