# Supplementary figures and images for: GRIN2A-related disorders: genotype and functional consequence predict phenotype
Source: Brain. 2018 Dec 12;142(1):80–92. doi: 10.1093/brain/awy304 (PMC6308310; doi:10.1093/brain/awy304)

**A** Hypotonia  
n = 139

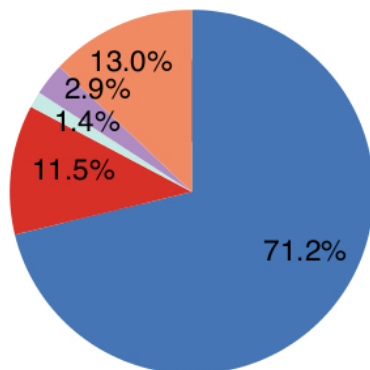

**B** Brain MRI  
n = 85

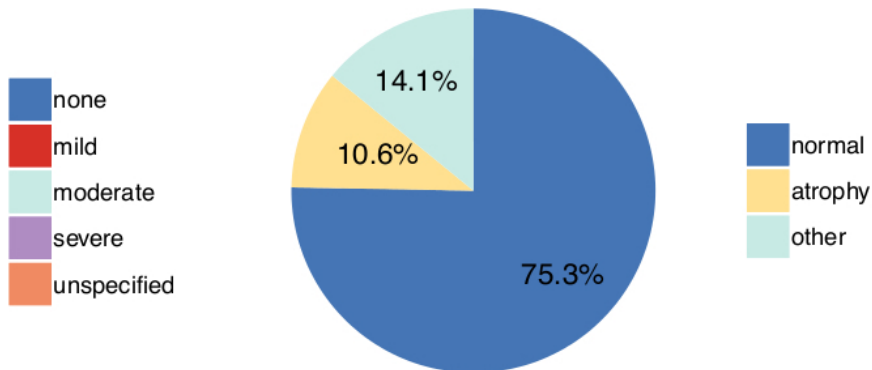

**C** Movement disorder  
n = 72

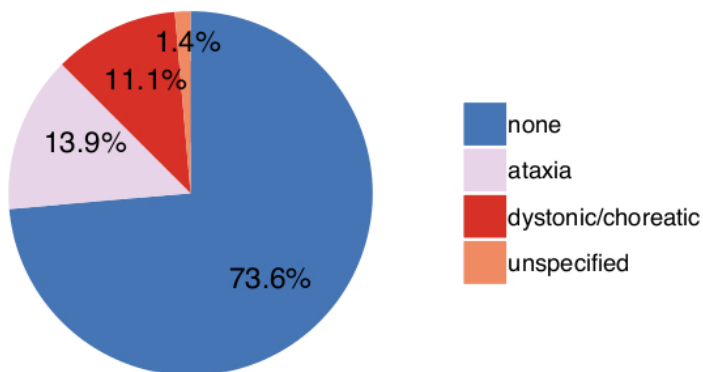

Supplement: Supplementary Data [file awy304_supp.zip › awy304-suppl_data/brain-2018-01122-File008.pdf]

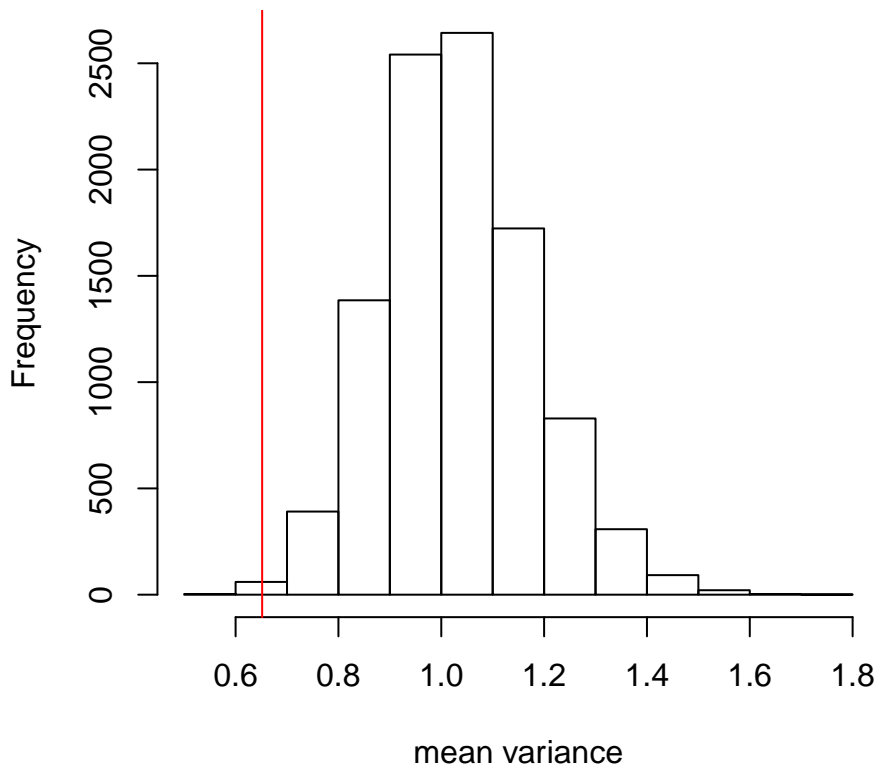

Supplement: Supplementary Data [file awy304_supp.zip › awy304-suppl_data/brain-2018-01122-File009.pdf]

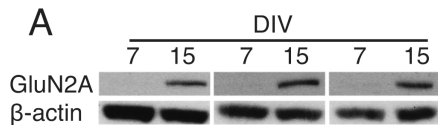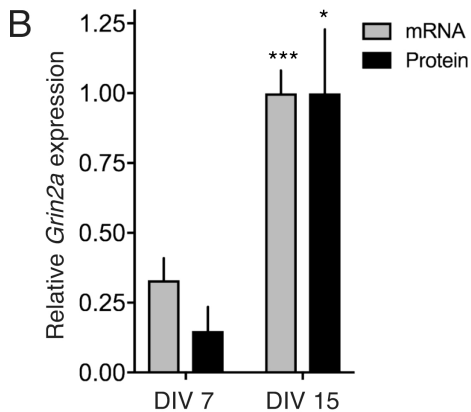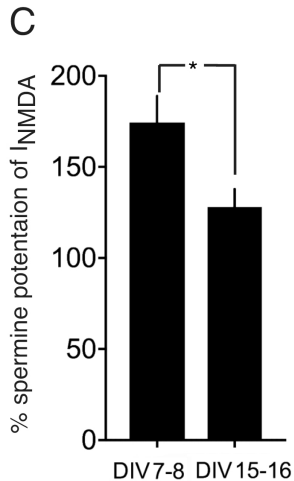

Supplement: Supplementary Data [file awy304_supp.zip › awy304-suppl_data/brain-2018-01122-File010.pdf]
